# Co-designing an integrated intervention package to strengthen adolescent sexual and reproductive health services in primary healthcare units in Southern Ethiopia: A human-centered design approach

**DOI:** 10.1371/journal.pgph.0007554

**Published:** 2026-11-11

**Authors:** Negussie Boti Sidamo, Mengistu Meskele, Sultan Hussen Hebo, Abinet Teshome, Bilcha Oumer, Yenus Ibrahim, Manaye Yihune Teshale, Getnet Mitike, Yalemzer Agegnehu

**Affiliations:** 1 Department of Public Health, College of Medicine and Health Sciences, Arba Minch University, Arba Minch, Ethiopia; 2 School of Public Health, College of Health Sciences and Medicine, Wolaita Sodo University, Wolaita Sodo, Ethiopia; 3 Department of Biomedical Sciences, College of Medicine and Health Sciences, Arba Minch University, Arba Minch, Ethiopia; 4 Department of Midwifery, Arba Minch Health Science College, Arba Minch, Ethiopia; 5 International Institute for Primary Health Care-Ethiopia, Addis Ababa, Ethiopia; University of St Andrews, UNITED KINGDOM OF GREAT BRITAIN AND NORTHERN IRELAND

## Abstract

Adolescent sexual and reproductive health (ASRH) remains a major public health priority in Ethiopia, where adolescents face barriers to accessing confidential, responsive, and high-quality sexual and reproductive health services. Although various ASRH interventions have been implemented in Ethiopia, limited involvement of adolescents and other stakeholders in participatory intervention development may constrain their contextual relevance, acceptability, and sustainability. This study applied Human-Centered Design (HCD) principles to co-design an integrated, adolescent-responsive intervention package for primary healthcare facilities in Southern Ethiopia. An intervention development study using an HCD approach was conducted from May to October 2025 in Arba Minch town and surrounding districts. The process followed five iterative HCD phases: empathize, define, ideate, prototype, and test/refine. A total of 61 participants, including adolescents, healthcare providers, facility managers, and community stakeholders, contributed through formative assessments, stakeholder workshops, problem identification and prioritization exercises, and co-design sessions. Qualitative data from interviews, discussions, and workshops were analyzed thematically, while participatory prioritization and iterative feedback informed intervention refinement. The process identified and prioritized 57 barriers affecting ASRH service delivery, access, and utilization. These findings informed the development of four complementary intervention components: strengthening healthcare provider and facility manager capacity through training, mentorship, and supportive supervision; establishing an adolescent peer navigator system to improve awareness, service uptake, access, and linkage to care; establishing a confidential sexual and reproductive health call center to provide counseling, appointment support, and referrals; and strengthening adolescent-responsive primary healthcare facilities through improved privacy, accessibility, service organization, and responsiveness to adolescents’ needs. Together, these components address barriers at the provider, facility, community, and service-access levels. The resulting package offers a contextually grounded, multilevel approach to integrating adolescent-responsive ASRH services into routine primary healthcare. By involving adolescents and key stakeholders throughout the design process, the study provides a foundation for developing services that better reflect adolescents’ needs and local health system realities. Further research is needed to evaluate the package’s feasibility, acceptability, implementation, and effectiveness, and to determine its potential for adaptation and scale-up in other settings.

## Introduction

Adolescence is a pivotal stage of development characterized by rapid physical, psychological, and social transitions that shape lifelong health and well-being [1]. Although the World Health Organization defines adolescence as 10–19 years, emerging evidence suggests that extending this range to 10–24 years better reflects the prolonged biological, psychological, and social transitions experienced by many young people [2]. This broader age range is particularly relevant in low-resource settings, where structural and socioeconomic factors including prolonged dependence on caregivers, delayed educational and economic transitions, early marriage, unemployment, and limited access to adolescent-responsive health services can extend vulnerability well into early adulthood [1,3,4]. Recognizing individuals aged 10–24 years as adolescents and young people therefore provides a more appropriate framework for designing and implementing interventions that address their evolving developmental and health needs [5,6].

Despite being a critical period for establishing lifelong health behaviors, young people in low- and middle-income countries continue to face persistent barriers to accessing SRH services [7–9]. Key challenges include limited availability of adolescent-responsive care, social stigma, lack of privacy, and fragmented service delivery [10–12]. In Ethiopia, specifically, where adolescents constitute over one-fifth of the population, these barriers are particularly pronounced, encompassing provider bias, insufficient privacy and confidentiality, limited access to contraceptives, and pervasive stigma surrounding adolescent sexuality [13–15]. Consequently, these barriers contribute to increased risks of unintended pregnancy, unsafe abortion, and sexually transmitted infections [16].

Conventional “top-down” ASRH interventions refer to programs that are primarily designed, planned, and implemented by experts, policymakers, or external implementing agencies with limited or no meaningful involvement of adolescents and other end-users in decision-making processes. Such approaches often emphasize predetermined priorities and standardized delivery models rather than co-created solutions grounded in the lived experiences of young people. Consequently, they have shown limited effectiveness in addressing the realities, preferences, and contextual needs of adolescents. This limitation is largely attributed to insufficient meaningful engagement of adolescents and other key stakeholders in the design and adaptation of interventions [17,18].

In Ethiopia, ASRH interventions have predominantly been expert-driven, with limited application of participatory design approaches [19]. In addition, the existing evidence base has largely focused on intervention effectiveness and service utilization outcomes, with insufficient attention to the extent to which adolescents and other key stakeholders are meaningfully engaged in the design and adaptation of interventions to ensure contextual relevance and responsiveness [20,21]. Collectively, these gaps underscore the limitations of conventional intervention approaches and highlight the need for more participatory, context-responsive, and implementation-oriented ASRH strategies.

To address the limitations of conventional top-down approaches, there is increasing recognition of the value of participatory and context-responsive approaches in health intervention design [22]. HCD is a structured and iterative approach to problem-solving that places the needs, preferences, experiences, and contextual realities of intended users at the center of the design process. It commonly involves iterative stages of understanding users’ experiences, defining problems, generating potential solutions, and refining promising options through engagement with intended users and relevant stakeholders [23–25]. Co-design can be incorporated within HCD as a collaborative process through which intended users and other stakeholders actively contribute to identifying and prioritizing problems, generating potential solutions, and refining these solutions based on their experiences and perspectives.

In this study, HCD provided the overarching framework for understanding barriers to adolescents’ access to and use of SRH services and for developing contextually relevant potential solutions. Co-design was used within this framework to engage adolescents, healthcare providers, facility managers, and other relevant stakeholders in the identification and prioritization of barriers and the generation and refinement of potential intervention components [26].This approach was particularly appropriate because ASRH service use is influenced by multiple individual, interpersonal, community, and health-system factors, which require solutions that are responsive to local needs and health-system realities. Although HCD is increasingly applied in health intervention development, empirical evidence on its application to ASRH programming in Ethiopia remains limited, particularly for developing integrated interventions within routine primary healthcare systems [25,27].

Therefore, this study used an HCD approach incorporating co-design to develop an integrated package of interventions to strengthen ASRH services within primary healthcare settings in Southern Ethiopia. By engaging adolescents and relevant health-system and community stakeholders throughout the design process, the study sought to develop contextually grounded and potentially feasible solutions that reflect users’ experiences and local health-system realities.

## Materials and methods

### Ethics statement

This study was conducted in accordance with the Declaration of Helsinki. Ethical approval was obtained from the Institutional Review Board (IRB) of Arba Minch University, College of Medicine and Health Sciences (Approval No: IRB/23339/25) on May 20^th^, 2025, prior to data collection. Written informed consent was obtained from all participants. For participants under 18 years, consent was obtained from parents or legal guardians, and assent was obtained from the adolescents. Participants aged 18 years and above provided their own written consent.

To ensure confidentiality, privacy, and anonymity, identifying information was removed from all reports, and participant identifiers were used only by the research team for internal purposes. Data collection occurred in private settings, participation was voluntary, and participants were informed of their right to withdraw at any time without consequences. All reported data were anonymized to prevent identification by family members, community members, or healthcare providers.

### Study setting

This study was conducted in Arba Minch town and surrounding districts of the Gamo Zone, Southern Ethiopia, encompassing both urban and rural communities served by primary healthcare (PHC) facilities. The PHC system comprises health centers and primary hospitals that provide essential SRH services, including family planning, HIV testing and counseling, antenatal care, and youth-friendly services. Despite these service platforms, adolescents continue to experience substantial barriers to accessing and utilizing SRH services, including limited adolescent-responsive service environments, inadequate privacy and confidentiality, insufficient provider competencies, periodic stock-outs of essential commodities, and sociocultural norms that discourage care-seeking [14]. These contextual challenges highlight the need for interventions that are grounded in the lived experiences of adolescents while remaining feasible within routine primary healthcare systems.

### Study period

The study was conducted from 2**5 May to 5 October 2025**.

### Study design

This study employed an **intervention development design using** HCD **approach incorporating co-design**. The HCD process was used to understand barriers to adolescents’ access to and use of SRH services and to develop contextually relevant potential solutions within primary healthcare settings. Co-design was incorporated as the collaborative process through which adolescents, healthcare providers, facility managers, and other relevant stakeholders contributed to the identification and prioritization of barriers, generation of potential solutions, and iterative refinement of intervention components.

The design followed five iterative HCD phases: **empathize, define, ideate, prototype, and test/refine**. These phases were implemented through formative assessment, stakeholder engagement, problem identification and prioritization, co-design workshops, and iterative refinement of the proposed intervention components. The process was intended to generate an integrated intervention package responsive to adolescents’ needs and aligned with the organizational and contextual realities of primary healthcare services in Southern Ethiopia.

Findings from the preceding formative assessment, which informed the HCD process, are reported separately [28].

## Participants and sampling

Stakeholder participants were purposively selected to ensure representation of key groups involved in, affected by, or able to influence the delivery and use of ASRH services in primary healthcare settings. The study included adolescents, healthcare providers, facility managers, district-level health officials, and community representatives. Adolescents were included as intended users of ASRH services and contributed perspectives based on their experiences, needs, and expectations, while healthcare providers and facility managers contributed perspectives on service delivery, operational constraints, and health-system realities. District-level health officials contributed health-system and implementation perspectives, and community representatives contributed contextual and community-level perspectives relevant to adolescents’ access to and use of ASRH services.

A purposive sampling approach was used to identify participants with relevant experiences, knowledge, or responsibilities related to ASRH services. Participant selection considered diversity of perspectives and roles rather than statistical representativeness. A total of 61 stakeholder participants took part in the HCD process, including 36 adolescents, 12 healthcare providers, 6 facility managers, 3 district-level health officials, and 4 community representatives. Participants contributed to different HCD activities according to their roles, experiences, and relevance to the specific stage of the design process.

Four researchers constituted the HCD facilitation team. They supported workshop planning and facilitation, documentation and synthesis of stakeholder inputs, and coordination of the iterative development and refinement of intervention components. The researchers contributed methodological, contextual, and implementation expertise but were not counted among the stakeholder participants. Intervention priorities were not determined independently by the research team; rather, they emerged through stakeholder discussions, prioritization exercises, iterative feedback, and consensus-building during the HCD process.

The composition of participants was intentionally designed to bring together the perspectives of intended service users and key health-system and community stakeholders. Stakeholder engagement was maintained across successive HCD phases to support continuity of learning and iterative refinement of the emerging integrated intervention package. This enabled the co-design process to integrate adolescents’ lived experiences with provider, managerial, health-system, and community perspectives when identifying and prioritizing barriers and developing and refining potential intervention components. The composition, recruitment approach, roles, and engagement of each stakeholder group across the HCD process are summarized in Table 1.

**Table 1:** Participant groups and sampling approach in the HCD co-design process.

Stakeholder group	No. of participants (n)	Recruitment/sampling approach	Role in the HCD co-design process	HCD phase(s)
**Adolescents and young people**	36	Purposive sampling based on relevant experience and perspectives as intended users of ASRH services	Shared lived experiences and needs; identified and prioritized barriers; generated potential solutions; provided feedback on emerging intervention components	Empathize, Define, Ideate, Prototype, Test/Refine
**Healthcare providers**	12	Purposive sampling based on experience in delivering ASRH/SRH services	Described service-delivery processes and constraints; identified implementation challenges; contributed to solution generation and refinement; assessed feasibility	Empathize, Define, Ideate, Prototype, Test/Refine
**Facility managers**	6	Purposive sampling based on facility management responsibilities and knowledge of service organization	Identified facility-level and operational constraints; assessed resource and organizational requirements; contributed to development and refinement of feasible intervention components	Define, Ideate, Prototype, Test/Refine
**District-level health officials**	3	Purposive sampling based on health-system planning, coordination, and implementation responsibilities	Provided health-system, policy, coordination, and implementation perspectives; assessed alignment with existing health-system structures and priorities	Define, Ideate, Prototype, Test/Refine
**Community representatives**	4	Purposive sampling based on community knowledge and involvement in adolescent and health-related issues	Contributed community and contextual perspectives; identified social and cultural considerations; assessed contextual relevance and acceptability of potential solutions	Define, Ideate, Prototype, Test/Refine
**Total stakeholder participants**	**61**	**Purposive sampling**	**Integrated stakeholder perspectives throughout the iterative HCD process**	**Across relevant HCD phases**
**HCD facilitation team (researchers)**†	**4**	Research team members	Planned and facilitated HCD activities; documented and synthesized stakeholder inputs; coordinated iterative refinement; provided methodological and technical guidance	All phases

HCD = human-centered design; ASRH = adolescent sexual and reproductive health; SRH = sexual and reproductive health. Stakeholder participants were purposively selected to provide diverse and relevant perspectives rather than statistical representativeness. The four researchers constituted the HCD facilitation team and were **not included in the 61 stakeholder participants**. Intervention priorities were generated through stakeholder discussions, prioritization exercises, iterative feedback, and consensus-building rather than being determined independently by the research team.

### Conceptual framing: Human-centered design and co-design

The study used HCD as the overarching methodological framework, incorporating co-design as the collaborative process for developing and refining potential intervention components. The HCD framework guided the iterative exploration of adolescents’ experiences and needs, identification and prioritization of barriers, generation of potential solutions, and refinement of intervention components in relation to the local primary healthcare context.

Within this framework, co-design activities engaged adolescents and relevant health-system and community stakeholders in identifying and prioritizing barriers, generating potential solutions, discussing alternative options, and providing iterative feedback on emerging intervention components. Stakeholder contributions were used to refine the proposed solutions according to their perceived relevance, acceptability, and potential feasibility.

Thus, in this study, HCD refers to the overall methodological approach, whereas co-design refers to the specific collaborative activities undertaken within that approach. This distinction was maintained throughout the study procedures and reporting.

## Human-centered design-guided co-design process

The intervention development process was guided by **HCD** and implemented through five interconnected phases: **Empathize, Define, Ideate, Prototype, and Test/Refine** (Fig 1). Each phase was operationalized through a structured co-design workshop, with stakeholder feedback and outputs from each workshop informing the subsequent phase. Thus, the HCD phases represent the methodological stages of the design process, whereas the workshops represent the structured activities through which each phase was implemented.

**Fig 1:**
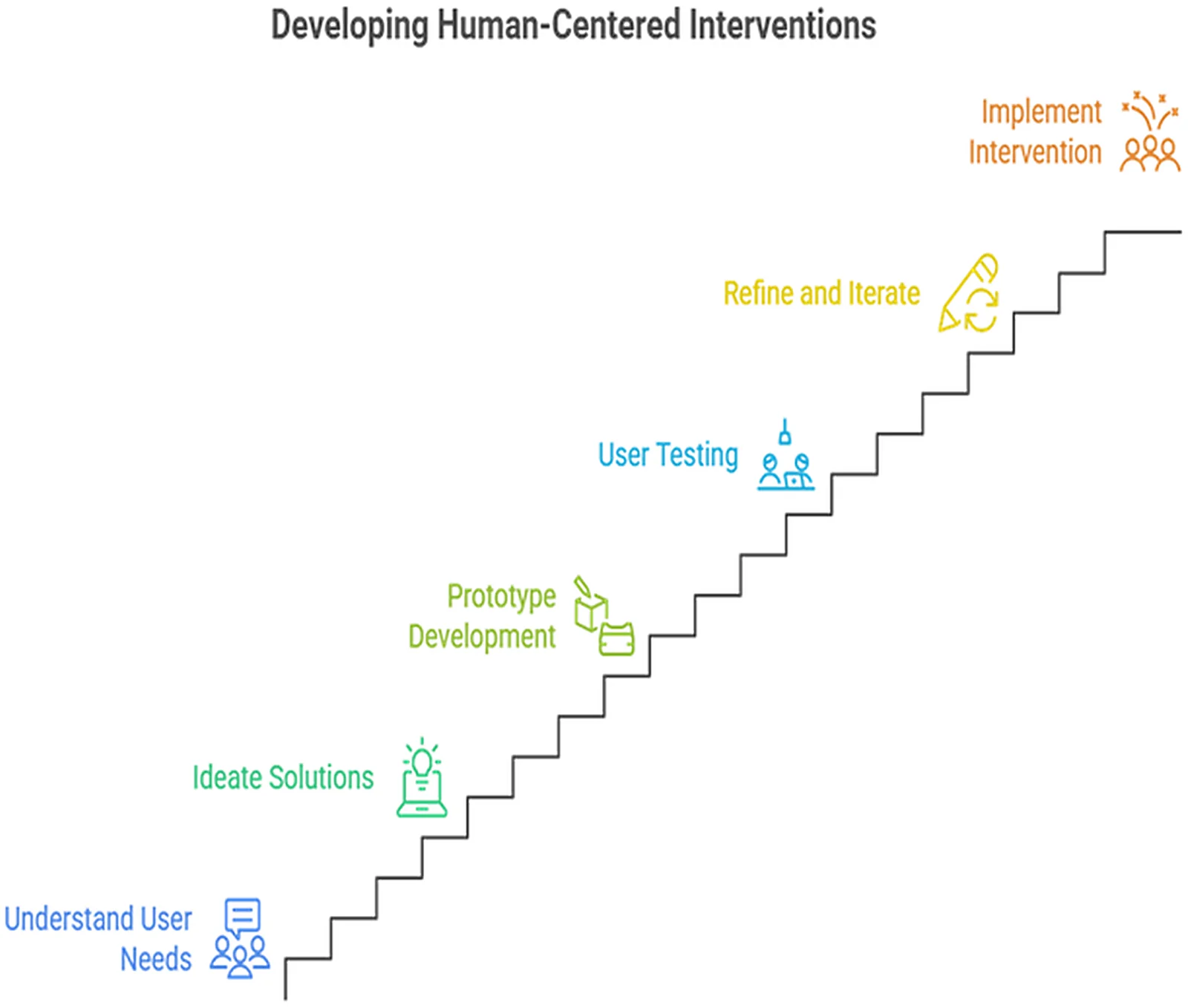
Human-centered design-guided co-design process for developing the integrated intervention package to strengthen adolescent sexual and reproductive health services in primary healthcare settings.

The five workshops were designed to progressively move from understanding stakeholder experiences and service barriers, through problem prioritization and solution generation, to prototype development, testing, and refinement of the integrated intervention package. The stakeholder groups, number of sessions, duration, main activities, and key outputs of each workshop are summarized in Table 2. A detailed overview of the human-centered design (HCD) co-design process used to develop the adolescent-responsive primary healthcare intervention in South Ethiopia is provided in S5 Appendix.

**Table 2:** Human-centered design phases, workshop structure, activities, and key outputs.

Workshop	HCD phase and purpose	Core activities	Stakeholder groups	Sessions	Duration	Key outputs
**Workshop 1**	**Empathize:** explore stakeholders’ experiences, needs, and barriers related to adolescent SRH services	Review of formative findings; exploration of adolescent experiences and service barriers; validation and contextualization of findings	Adolescents/young people; healthcare providers; facility managers; district health officials; community representatives	2	6 h 20 min	Validated and consolidated barriers; contextual understanding of ASRH service challenges
**Workshop 2**	**Define:** synthesize, prioritize, and frame key problems	Synthesis of Empathize findings; affinity mapping; problem definition; weighted MCDA prioritization; formulation of “How might we?” (HMW) questions	Adolescents/young people; healthcare providers; facility managers; district health officials; community representatives	2	6 h 50 min	Thematic clusters; prioritized ASRH problems; HMW questions
**Workshop 3**	**Ideate:** generate and prioritize potential solutions	Individual and group ideation; solution development and discussion; idea clustering; feasibility–impact assessment; plenary prioritization	Adolescents/young people; healthcare providers; facility managers; district health officials; community representatives	2	7 h 20 min	Consolidated and prioritized intervention concepts
**Workshop 4**	**Prototype:** develop, review, and refine intervention prototypes	Development of preliminary prototypes; review of intervention components; identification of implementation requirements; stakeholder feedback; prototype refinement; preparation of pilot-ready components	Adolescents/young people; healthcare providers; facility managers; district health officials; community representatives	2	7 h 40 min	Refined intervention prototypes; pilot-ready intervention components
**Workshop 5**†	**Test/Refine:** assess implementation experience and identify required adaptations	Review of pilot experiences; identification of implementation challenges; assessment of acceptability, usability, feasibility, and contextual fit; identification and prioritization of adaptations	Adolescents/young people; healthcare providers; facility managers; district health officials; community representatives	1	1 day	Stakeholder feedback; refinement priorities; agreed adaptations

† Workshop 5 was the participatory feedback workshop conducted within the broader Test/Refine phase. The Test/Refine phase also included implementer training, pilot implementation, supportive supervision, intervention refinement, and finalization of the integrated intervention package.

### Empathize phase – Workshop 1

The **Empathize phase** focused on developing a contextual understanding of adolescents’ experiences, needs, and barriers related to accessing and using ASRH services. Formative evidence generated through the study was presented and discussed with stakeholders. Participants explored adolescent experiences, service-delivery challenges, unmet needs, and contextual factors affecting access to and utilization of ASRH services.

Stakeholder discussions were used to validate and enrich the formative findings. Similar barriers were identified and consolidated, while distinct barriers were retained where they represented different experiences, mechanisms, or potential intervention targets. The outputs of Workshop 1 provided the evidence base for the Define phase.

### Define phase – Workshop 2

The Define phase focused on synthesizing the findings from the Empathize phase into clearly defined and prioritized implementation challenges affecting ASRH service delivery and utilization. During Workshop 2, adolescents and young people, healthcare providers, facility managers, district health officials, community representatives and researchers jointly reviewed and interpreted the consolidated formative evidence. Related barriers were grouped into thematic clusters and subsequently translated into specific problem statements, considering their relevance to adolescents, service providers, facilities, and the broader health-system context.

To systematically prioritize the identified challenges, a weighted multi-criteria decision analysis (MCDA) was applied. The MCDA provided a transparent and reproducible approach for assessing challenges across multiple dimensions relevant to intervention development and implementation within routine PHC settings. Five predefined criteria were used: impact/severity (30%), equity (25%), feasibility (20%), reach/magnitude (15%), and urgency (10%). The criteria were informed by the study objectives, formative findings, HCD principles, and practical considerations for adolescent health and routine PHC delivery. Their relevance and contextual appropriateness were reviewed with participating stakeholders before prioritization, and the criterion weights were normalized to sum to 100%.

Each implementation challenge was scored on a 1–5 ordinal scale for each criterion using predefined operational definitions and scoring anchors. Higher scores indicated greater impact/severity, equity concern, reach/magnitude, and urgency; for feasibility, higher scores indicated greater practicality and achievability within the existing health-system context. A weighted priority score was calculated for each challenge by multiplying the criterion-specific score by its corresponding weight and summing across the five criteria. Because the weights summed to 1.0, the resulting composite score ranged from 1 to 5, with higher scores indicating greater overall priority. Challenges were then ranked according to their composite scores.

The prioritized challenges were subsequently synthesized and reframed as user-centered **“How Might We” (HMW)** questions. This reframing translated priority implementation problem into actionable design opportunities and provided structured design prompts for the **Ideate phase**. The outputs of Workshop 2 therefore included **consolidated thematic clusters, prioritized ASRH problem statements, and HMW questions** that guided subsequent solution generation. The complete MCDA framework, including criterion definitions, scoring anchors, weights, and the scoring and ranking matrix, is provided in S2 Appendix.

### Ideate phase – Workshop 3

The **Ideate phase** focused on generating, organizing, and prioritizing potential solutions to the prioritized **“**How Might We” (HMW) questions developed during the Define phase. **Adolescents and young people, healthcare providers, facility managers, district health officials, and community representatives** participated in Workshop 3. The HMW questions were first reviewed to establish a shared understanding of the priority challenges and design opportunities. Participants then generated intervention ideas individually before sharing them within facilitated thematic groups. Individual idea generation was used to encourage diverse perspectives and reduce the potential influence of dominant voices during the initial stage of ideation. Within the thematic groups, participants discussed, combined, refined, and expanded the proposed solutions.

The generated ideas were subsequently organized using **affinity mapping**. Similar or conceptually related ideas were grouped, overlapping or duplicate ideas were consolidated, and related ideas were synthesized into broader intervention themes. This process reduced conceptual duplication while preserving distinct intervention approaches. The resulting themes and shortlisted ideas were then subjected to a structured **Feasibility–Impact Assessment** to identify intervention concepts suitable for further development and prototyping within routine PHC settings.

Each thematic group assessed its shortlisted intervention ideas using a **Feasibility–Impact Matrix**. **Feasibility** was assessed in relation to technical simplicity, availability of required resources, cost-effectiveness, and compatibility with existing policies, programs, and health-system structures. **Potential impact** was assessed according to the anticipated contribution to SRH outcomes, equity, service coverage, and sustainability. Participants assigned each idea a score from **1 (low) to 5 (high)** for both feasibility and potential impact. The scores were plotted on a two-dimensional matrix to facilitate comparison and identify concepts with a favorable combination of feasibility and potential impact.

The preliminary prioritization was subsequently reviewed during a **plenary session**. Participants discussed the results, clarified differences in interpretation, considered contextual and implementation requirements, and reached agreement on the intervention concepts to be advanced to the Prototype phase. Thus, prioritization combined **structured quantitative scoring with participatory deliberation**, ensuring that selection of intervention concepts was informed not only by their feasibility and potential impact scores but also by stakeholder knowledge of the local context and routine PHC implementation environment.

The primary output of Workshop 3 was a **consolidated and prioritized set of intervention concepts** that responded to the priority ASRH problems and HMW questions identified during the Define phase and were considered sufficiently feasible and potentially impactful to proceed to prototyping.

### Prototype phase – Workshop 4

During Workshop 4 (Prototype phase), intervention concepts prioritized during the Ideate phase were translated into low-fidelity prototypes representing the core features, processes, interactions, and implementation requirements of the proposed intervention package. Depending on the intervention concept, prototypes included service-delivery workflows, referral pathways, provider-support components, communication materials, and role-play scenarios.

Prototypes were developed collaboratively with relevant stakeholders and subjected to iterative review through facilitated group discussions, simulation exercises, and structured review of usability and implementation requirements. Participants examined each prototype in relation to its intended use within routine PHC settings and identified potential barriers, ambiguities, operational gaps, and implementation requirements. The review focused on feasibility, acceptability, usability, contextual appropriateness, and compatibility with existing PHC workflows, roles, and systems.

Feedback generated during the workshop was documented, consolidated, and used to iteratively modify the prototypes. Key elements were simplified, clarified, reorganized, or adapted where necessary to improve usability, operational clarity, and fit with routine service delivery. Revised prototypes were reviewed collaboratively to ensure alignment with the priority ASRH problems and “How Might We” (HMW) questions established during the Define phase.

The primary output of Workshop 4 was a set of refined low-fidelity intervention prototypes and pilot-ready intervention components, which were subsequently advanced to the Test/Refine phase for further assessment under routine PHC conditions.

### Test/Refine phase – Workshop 5

The Test/Refine phase focused on assessing the feasibility, usability, acceptability, and contextual fit of the prototype intervention package under routine PHC conditions. Following prototype development, implementers participated in a three-day training covering the intervention components, implementation procedures, roles and responsibilities, and practical application of the package. The intervention was subsequently piloted in four purposively selected PHC facilities.

During the pilot, the research team provided **supportive supervision** to document implementation processes, identify operational challenges, assess the usability of intervention components, and identify adaptations required for integration into routine service delivery. Supervision focused on implementation experience and contextual fit rather than estimating intervention effectiveness. Implementation experiences and emerging operational issues were documented throughout the pilot to inform subsequent refinement.

Following the pilot implementation, Workshop 5, a participatory feedback workshop was conducted with adolescents and young people, healthcare providers, facility managers, district health officials, and community representatives. Participants reflected on their experiences with the intervention and provided structured feedback regarding its acceptability, usability, feasibility, contextual appropriateness, and compatibility with existing PHC workflows and service-delivery processes. Participants also identified implementation barriers, enabling factors, and components requiring further modification or support.

Feedback from the pilot implementation, supportive supervision, and Workshop 5 was systematically reviewed and synthesized by the research team in collaboration with stakeholders. Identified issues were used to iteratively modify the intervention package, including clarifying or simplifying procedures and adjusting implementation processes where necessary. Refinements were made while preserving the core functions and intended mechanisms of the intervention components, thereby maintaining fidelity to the underlying intervention design while improving contextual fit and implementability.

Across the HCD process, researchers primarily served as **facilitators and technical advisors**, supporting evidence synthesis, workshop facilitation, documentation of design decisions, and methodological oversight. Standardized facilitation guides and structured participatory techniques were used to promote consistency across workshops, encourage diverse perspectives, and support transparent decision-making. Detailed facilitator guides, workshop agendas, activity instructions, guiding questions, and facilitation prompts are provided in S1 Appendix.

Although the HCD phases are presented sequentially for clarity, they were implemented as an **iterative and interconnected process** rather than as strictly linear stages. Stakeholder perspectives, co-design outputs, implementation experiences, and emerging evidence were revisited across successive phases to refine priorities, intervention concepts, prototypes, and implementation processes. The overall process therefore progressed from formative evidence and problem identification, through problem prioritization and solution generation, to prototyping, testing, and iterative refinement, culminating in the final integrated intervention package for strengthening adolescent-responsive ASRH service delivery within routine PHC settings.

## Data collection procedures

All in-depth interviews and co-design workshops were conducted in the local language by trained qualitative researchers with demonstrated expertise in adolescent health research and participatory HCD methodologies. This ensured linguistic appropriateness, cultural sensitivity, and methodological consistency across all data collection activities. With prior informed consent, all sessions were audio-recorded to ensure accurate and complete capture of participant narratives. Recordings were transcribed verbatim, and where necessary, translated into English while maintaining conceptual and contextual equivalence to preserve meaning and analytic integrity.

Comprehensive field notes were systematically maintained throughout all sessions to document non-verbal communication, group dynamics, contextual influences, and reflexive observations of the research team. These field notes complemented audio-recorded data by capturing interactional, behavioral, and situational dimensions that were not fully represented in transcripts. To strengthen methodological rigor, credibility, and triangulation, multiple complementary qualitative data sources were collected.

These included facilitator notes, flip charts, visual outputs, and a range of workshop artifacts generated during participatory activities. The integration of these diverse data streams enabled a more nuanced and robust interpretation of findings, enhancing analytic depth, validity, and trustworthiness.

### Data analysis

Data were analyzed using an inductive thematic analysis approach embedded within an iterative HCD co-design process to generate contextually relevant insights for intervention development [29]. HCD was used as a participatory intervention development framework rather than as an analytical coding framework. Qualitative analysis focused on identifying patterns, meanings, experiences, needs, barriers, facilitators, and opportunities emerging from participants’ accounts, workshop discussions, and participatory design artifacts. Analysis was conducted iteratively and concurrently with data collection, allowing emerging findings to inform subsequent co-design activities and progressive refinement of intervention components.

Qualitative data sources included in-depth interview transcripts, co-design workshop transcripts, field notes, observational records, facilitator reflections, and participatory design artifacts generated throughout the HCD process. Analysis began with repeated reading of transcripts and supporting materials to achieve data familiarization. Two researchers independently conducted line-by-line inductive coding using ATLAS.ti version 8 .Codes were generated directly from the data without applying predefined HCD categories. Researchers continuously compared codes across participant groups and data sources using the constant comparative approach, with iterative refinement through regular analytical discussions until consensus was achieved.

Following inductive coding, related codes were organized into broader categories and preliminary themes through an iterative process of interpretation and team discussion. The resulting themes represented key user experiences, implementation barriers, enabling factors, service delivery challenges, and intervention needs identified by participants. Subsequently, HCD techniques such as affinity mapping were applied during co-design workshops to support participatory synthesis and translate research findings into intervention design requirements. This process enabled stakeholders to collectively interpret findings, prioritize challenges, and identify potential solutions.

The synthesized findings informed subsequent HCD activities. Priority challenges identified through thematic analysis were reviewed and validated with stakeholders during the Define phase and transformed into user-centered problem statements. These statements were further reframed as “How Might We" (HMW) questions, which guided collaborative ideation, intervention prioritization, prototype development, and iterative refinement of intervention components.

Methodological triangulation was achieved through systematic comparison of findings across multiple data sources, including interviews, workshops, observations, and participatory design artifacts. Emerging interpretations and intervention concepts were reviewed with stakeholders during subsequent co-design sessions for validation and refinement, enabling clarification of divergent perspectives and collaborative consensus-building. An audit trail documenting coding decisions, analytical interpretations, design modifications, and consensus-building processes was maintained throughout the study to enhance transparency, dependability, and reproducibility.

## Reflexivity and researcher positionality

The research team comprised public health researchers and practitioners with expertise in ASRH, primary health care, implementation research, and Human-Centered Design. These professional backgrounds and prior experience with adolescent health and PHC services may have shaped how participants’ experiences were interpreted and how intervention ideas were facilitated and prioritized. The research team therefore recognized the potential for their professional authority, assumptions, and familiarity with the health system to influence participant responses and the co-design process.

To minimize potential power imbalances and facilitator influence, facilitators used standardized discussion guides and structured HCD techniques and avoided introducing predetermined intervention solutions. Participants were encouraged to generate and articulate ideas in their own words, while facilitators used open-ended probing to explore different and divergent perspectives rather than directing participants toward consensus. Intervention ideas were prioritized using predefined criteria applied consistently across proposed solutions, and the rationale for prioritization and subsequent refinement was documented.

Particular attention was given to ensuring that adolescents’ perspectives were not overshadowed by health professionals, facility managers, or other adult stakeholders. Facilitators actively encouraged adolescents and less vocal participants to contribute and created opportunities for adolescent perspectives to be expressed during the co-design activities. Adolescent-generated priorities, concerns, and proposed solutions were retained during synthesis and considered alongside the perspectives of professional stakeholders. Throughout the iterative HCD process, the research team reflected on facilitator assumptions, participant interactions, and potential sources of influence, using these reflections to inform subsequent facilitation, interpretation, and refinement of the intervention package.

## Results

### Characteristics of co-design participants and process overview

A total of 61 stakeholders participated in the HCD co-design process: 36 adolescents and young people, 12 healthcare providers, 6 health facility managers, 3 district health officials, and 4 community representatives (Table 3). Across the five HCD phases, the process progressed from identification and prioritization of ASRH service barriers to solution generation, prototype development, and iterative refinement.

**Table 3:** Stakeholder groups engaged in the co-design process and their contributions to intervention development in Southern Ethiopia.

Stakeholder group	Participants (n)	Perspective contributed	Contribution to intervention decisions
Adolescents and young people	36	Lived experiences of accessing SRH services, perceived barriers, unmet needs, preferences, and expectations for adolescent-responsive care	Identified priority challenges affecting SRH access; shaped intervention features related to confidentiality, respectful care, information access, peer support, and adolescent-friendly service delivery
Healthcare providers	12	Frontline experiences of delivering adolescent SRH services, provider–adolescent interactions, service delivery constraints, and opportunities within routine PHC practice	Informed provider-focused strategies, including capacity strengthening, supportive supervision, improved communication, and integration of adolescent-responsive approaches into routine care
Health facility managers	6	Facility-level readiness, operational requirements, resource implications, and service organization considerations	Guided adaptation of intervention components to facility realities, including workflow integration, privacy improvements, referral mechanisms, and implementation requirements
District health officials	3	Health-system priorities, policy alignment, implementation feasibility, and sustainability considerations	Ensured intervention alignment with local health priorities and informed strategies for system-level support and scale-up potential
Community representatives	4	Community norms, social influences, cultural acceptability, and contextual factors affecting adolescent SRH service use	Shaped approaches to improve community acceptability, address stigma, and strengthen supportive environments for adolescents

### Identification and characterization of ASRH service barriers

During the Empathize phase, stakeholders identified a broad range of barriers affecting adolescents’ access to and utilization of SRH information and services within PHC. Across stakeholder groups, limited utilization was perceived as resulting from the interaction of individual and interpersonal factors, family and community influences, and health-system and structural constraints, rather than from the availability of services alone.

Through stakeholder review and consolidation, 57 unique barriers were identified. Overlapping or conceptually similar findings were merged, while barriers representing distinct mechanisms or potential intervention targets were retained. The resulting barriers were organized into three interconnected domains: (1) individual and interpersonal barriers, (2) family and community barriers, and (3) health-system and structural barriers (S3 Appendix).

**Individual and interpersonal barriers** included limited knowledge and awareness of available SRH services, misconceptions regarding SRH and contraceptive methods, limited access to reliable information, fear of judgment and stigma, embarrassment or shame associated with service utilization, concerns about confidentiality, limited self-confidence, financial constraints, and restricted autonomy in decision-making.

**Family and community barriers** included limited parent–adolescent communication, unsupportive family environments, fear of negative family reactions, family disapproval, stigma surrounding adolescent SRH service use, restrictive social and gender norms, taboos surrounding adolescent sexuality, fear of exposure or name-calling, limited social support, and cultural or religious opposition to contraceptive use and SRH education.

**Health-system and structural barriers** included inadequate provider competency and training, judgmental or stigmatizing provider attitudes, concerns about confidentiality, inadequate privacy and adolescent-responsive spaces, age-related restrictions, inconvenient service hours, long waiting times, geographic and transportation barriers, commodity stockouts, inadequate adolescent-responsive information materials, weak referral and linkage mechanisms, inconsistent application of adolescent-responsive protocols, limited outreach, weak governance and intersectoral coordination, inadequate monitoring and accountability, and gaps in policy implementation, institutional commitment, and financing.

The barriers were not viewed as isolated obstacles but as interconnected and mutually reinforcing constraints. For example, concerns about confidentiality were compounded by perceived provider judgment and community stigma, discouraging adolescents from seeking facility-based services even when services were available. Similarly, limited access to reliable information contributed to misconceptions about SRH and available services, reduced confidence in seeking care, and increased reliance on peers and informal sources. At the service-delivery level, provider capacity and organizational constraints further reinforced concerns about privacy, confidentiality, and the responsiveness of available services. These findings indicated the need for a multilevel and integrated intervention approach rather than isolated strategies targeting individual barriers.

### Define phase: Prioritization of problems and HMW questions

Following identification and consolidation of the 57 barriers, stakeholders prioritized the challenges according to their importance for adolescent SRH and their relevance to intervention development within routine PHC settings. This process resulted in **19 high-priority problems** considered sufficiently important and actionable to warrant further intervention development.

The 19 priority problems were subsequently reframed into **10 HMW questions**, translating identified challenges into design-oriented opportunities for solution generation. The HMW questions focused on opportunities to strengthen adolescent-responsive service delivery, improve access to reliable SRH information and service navigation, and create more supportive environments for adolescent SRH care-seeking.

Stakeholder prioritization indicated strong interest in approaches that could address service-delivery barriers through existing PHC structures while also improving adolescents’ access to information and support. Participants recognized that family and community factors were important determinants of service utilization but also identified their dependence on broader and longer-term social and behavioral processes. The resulting HMW questions therefore provided a structured bridge between problem prioritization and solution generation in the Ideate phase.

### Ideate phase: Generation and prioritization of intervention concepts

In response to the 10 HMW questions, stakeholders generated **140 potential intervention ideas** during the Ideate phase. The ideas addressed barriers across multiple levels, including adolescent support and behavior, provider practices, service organization, community engagement, information provision, referral and linkage, and broader health-system strengthening.

Through affinity mapping and thematic consolidation, the 140 ideas were organized into three broad domains: service delivery strengthening (71 ideas), family and community engagement (40 ideas), and information and education (29 ideas). Service-delivery ideas emphasized provider competency, confidentiality, adolescent-responsive care, referral mechanisms, and integration of SRH services within routine PHC. Family and community ideas focused on reducing stigma, strengthening parental and community support, and creating enabling social environments. Information and education ideas included peer education, community outreach, school-based activities, and digital communication approaches.

The consolidated ideas were subsequently assessed using the **Feasibility–Impact Matrix** and reviewed through plenary deliberation. Stakeholders prioritized concepts that combined potential to address important ASRH barriers with practical feasibility within routine PHC settings. The prioritization process resulted in a set of complementary intervention concepts addressing provider and facility capacity, adolescent support and navigation, confidential access to SRH information and referral, and adolescent responsiveness of routine PHC services.

### Prototype phase: Development and refinement of intervention components

The prioritized intervention concepts were translated into low-fidelity prototypes to examine how they could function within routine PHC settings. Before prototyping, the clustered intervention ideas were assessed using predefined criteria, including feasibility, potential impact, acceptability, cultural appropriateness, sustainability, and compatibility with existing PHC systems and resources. Through facilitated stakeholder discussion and consensus building, solutions perceived as both potentially effective in addressing the prioritized barriers and feasible within routine PHC delivery were selected for prototyping.

The initial prototypes included provider capacity-building strategies, adolescent-responsive service workflows, peer navigation pathways, facility-level service adaptations, and confidential communication and referral mechanisms. Stakeholders examined these prototypes through role-play simulations, service-flow mapping and review, and facilitated feedback, focusing on usability, acceptability, contextual appropriateness, workflow integration, and clarity of roles and responsibilities. Feedback was used iteratively to simplify and clarify procedures, refine roles, adjust service-delivery processes, strengthen communication and referral pathways, and improve coordination across components.

The prototyping process reinforced that the barriers identified during the earlier phases were interconnected and operated across multiple levels. Stakeholders therefore considered a single intervention insufficient to address the range of factors influencing adolescent SRH service access and utilization. Instead, they supported an integrated package combining strategies to strengthen provider capacity, improve facility responsiveness, support adolescent service navigation, and expand confidential access to SRH information and referral services.

This iterative process of prioritization, prototyping, stakeholder testing, and refinement resulted in a set of complementary intervention components designed for integration within routine PHC services. The final intervention package reflected a balance between addressing priority adolescent SRH barriers and ensuring practical feasibility, acceptability, contextual appropriateness, and compatibility with existing PHC systems.

### Test/Refine phase: Pilot experience and iterative refinement

The refined prototype package was subsequently piloted in four purposively selected PHC facilities following a three-day training of implementers. The pilot provided an opportunity to examine how the intervention components functioned under routine service-delivery conditions and to identify practical implementation challenges that could not be fully anticipated during the earlier design and prototyping phases. Supportive supervision was conducted throughout implementation to document operational challenges, implementation requirements, and opportunities to improve the usability and contextual fit of the intervention components.

A participatory feedback workshop subsequently brought together adolescents, healthcare providers, facility managers, district health officials, and community representatives to reflect on their experiences with implementation. Discussions examined how the intervention functioned in practice, including its feasibility, acceptability, usability, contextual appropriateness, and compatibility with existing PHC workflows. Stakeholders also identified specific components and implementation procedures that required further modification.

Findings from the pilot, supportive supervision, and stakeholder feedback were synthesized and used to guide iterative refinement of the intervention package. Adaptations included clarifying and simplifying procedures, refining roles and implementation processes, and strengthening linkages between intervention components. Importantly, refinements were made while preserving the core functions and intended mechanisms of the intervention, thereby maintaining fidelity to the underlying design while allowing appropriate adaptation to the local PHC context.

The Test/Refine phase therefore provided an important final opportunity to validate the practical implementation of the intervention package and resolve context-specific challenges before subsequent implementation and evaluation. The resulting package incorporated lessons from both stakeholder co-design and real-world pilot experience, with improved alignment to the needs of adolescents, implementers, and routine PHC service-delivery conditions.

## Final integrated intervention package

The iterative process of co-design, prioritization, prototyping, pilot testing, and refinement culminated in a final integrated intervention package comprising four complementary components designed to address the interconnected barriers affecting adolescents’ access to and utilization of SRH services within routine PHC settings (Fig 2). Rather than establishing a parallel adolescent health service, stakeholders emphasized strengthening existing PHC structures by addressing interconnected barriers related to adolescent awareness and trust, provider capacity, facility responsiveness, and linkage to care. The four components were therefore designed to operate synergistically, with each addressing a distinct but related dimension of adolescent SRH access and service utilization.

**Fig 2:**
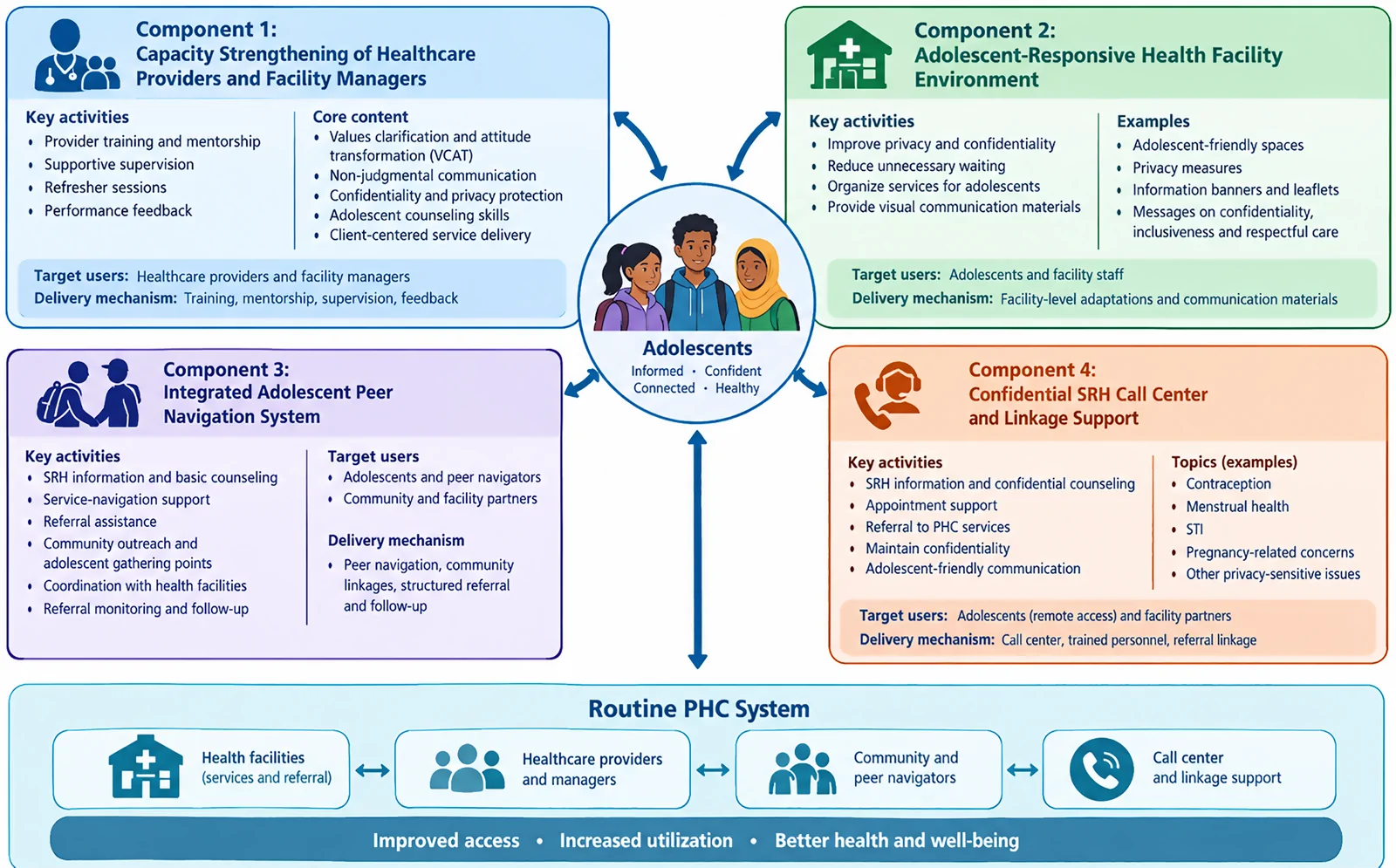
Co-designed adolescent-responsive primary health care intervention package in Southern Ethiopia.

### Component 1: Capacity strengthening of healthcare providers and facility managers

Stakeholders identified provider attitudes, limited adolescent-responsive competencies, and concerns about confidentiality as important barriers to adolescents’ willingness to seek and continue using SRH services. The first component therefore focused on strengthening healthcare providers’ and facility managers’ capacity to deliver respectful, confidential, non-judgmental, and adolescent-responsive care.

The component incorporated provider training, mentorship, supportive supervision, refresher sessions, and structured performance feedback. Core content included values clarification and attitude transformation (VCAT), non-judgmental communication, confidentiality and privacy protection, adolescent counseling skills, and client-centered service delivery. These activities were intended not only to improve providers’ knowledge and technical skills but also to strengthen the quality of adolescent–provider interactions and build adolescents’ trust in PHC services.

### Component 2: Adolescent-responsive health facility environment

Stakeholders reported that adolescents could perceive PHC facilities as intimidating environments in which privacy was uncertain and services were not sufficiently adapted to their needs. The second component therefore focused on practical improvements to the organization, physical environment, and communication systems of PHC facilities.

Key adaptations included measures to improve privacy, reduce unnecessary waiting, and organize services in a more adolescent-responsive manner. Visual communication materials, including banners and educational leaflets, were incorporated to increase awareness of available SRH services and communicate messages related to confidentiality, inclusiveness, and respectful care. These adaptations complemented provider capacity strengthening by recognizing that adolescent-responsive care depends not only on provider behavior but also on how services are organized and experienced within the facility.

### Component 3: Integrated adolescent peer navigation system

Limited awareness of available services, uncertainty about where to seek care, and concerns about approaching health facilities independently were identified as important barriers to SRH service utilization. The third component therefore established an integrated peer navigation system to connect adolescents with trusted information, counseling, and facility-based services. Trained adolescent peer navigators were designed to provide SRH information, basic counseling, service-navigation support, and referral assistance through community outreach, adolescent gathering points, and facility-linked activities. Structured referral pathways connected adolescents with appropriate PHC services and other available support mechanisms. Coordination with health facilities and monitoring of referrals were incorporated to strengthen follow-up, reduce loss to linkage, and support continuity of care. Peer navigation thus served as a bridge between adolescents and the formal health system by addressing barriers related to trust, information, navigation, and access.

### Component 4: Confidential SRH call center and linkage support

To address concerns about privacy, stigma, and difficulties obtaining reliable SRH information, stakeholders co-designed a confidential SRH call center and linkage support mechanism as an additional access pathway. The proposed service was intended to enable adolescents to obtain SRH information and confidential counseling remotely, receive appointment support, and obtain referral linkage to PHC services. Trained personnel were expected to use adolescent-friendly communication approaches, maintain confidentiality, and provide standardized SRH information, with referral linkages intended to support connection with facility-based services.

During the co-design process and early implementation, stakeholders identified demand for confidential information on contraception, menstrual health, sexually transmitted infections, pregnancy-related concerns, and other privacy-sensitive SRH questions. Participants described remote access as a potentially acceptable option because it could allow adolescents to seek information confidentially before deciding whether to visit a health facility. Facility stakeholders similarly perceived the call center as a potentially useful mechanism for facilitating earlier contact with adolescents and strengthening linkage to routine PHC services.

These findings reflect stakeholder perspectives and observations during the co-design and early implementation phases rather than evidence of effectiveness. The study did not formally evaluate call-center utilization, acceptability, referral completion, or health outcomes attributable to the call-center component.

### Integration of the four components

The four components were designed as an interconnected package rather than as stand-alone interventions. Provider capacity strengthening and facility-level adaptations addressed the supply-side responsiveness of PHC services by improving provider competencies, service organization, privacy, and the adolescent experience of care. Peer navigation and confidential communication and referral mechanisms addressed access, trust, information, and linkage to care, strengthening the pathways through which adolescents could reach and remain connected to appropriate services.

This configuration reflected stakeholders’ recognition that the barriers identified during the design process were interconnected and could not be adequately addressed through a single intervention. The final package therefore combined strategies operating at the provider, facility, adolescent, and communication/referral levels, while embedding these functions within existing PHC structures rather than creating a parallel service-delivery system.

The final intervention package thus represented the cumulative output of the design process, moving from barrier identification and prioritization through HMW framing, ideation, prototyping, pilot testing, and iterative refinement. Each component was refined to enhance its contextual relevance, feasibility, acceptability, and compatibility with routine PHC delivery while preserving its core function and intended mechanism. Detailed descriptions of the four components of the final integrated intervention package, including their objectives, key activities, delivery mechanisms, and implementation responsibilities, are provided in S4 Appendix. Fig 2 illustrates the final intervention package and the interrelationships among its four complementary components, highlighting how provider capacity strengthening, an adolescent-responsive facility environment, peer navigation, and confidential SRH call-center and linkage support were designed to address interconnected barriers to adolescents’ access to and utilization of SRH services within routine PHC settings.

## Discussion

This study used HCD approach to co-design and refine an integrated intervention package to strengthen ASRH services within routine PHC in Southern Ethiopia. Five findings are particularly important. First, barriers to adolescent SRH service access and utilization were interconnected and occurred across multiple levels, indicating the need for a multicomponent rather than single-intervention response. Second, stakeholders considered provider responsiveness including attitudes, communication, confidentiality, and counseling as an important determinant of adolescents’ experience of care. Third, mechanisms that improve access to information, trust, and service navigation were considered important for connecting adolescents with formal health services. Fourth, the organization and responsiveness of routine PHC facilities were viewed as important for creating an environment in which adolescents could seek and receive care. Finally, the iterative HCD process enabled adolescents, providers, managers, and other stakeholders to identify context-specific challenges and refine the intervention before subsequent implementation and evaluation. Together, these findings suggest that improving adolescent SRH services requires attention to both the demand-side and supply-side barriers that shape the pathway from seeking care to receive appropriate services. Importantly, this study did not establish the effectiveness of the resulting intervention package; rather, it demonstrates how participatory design and iterative refinement can be used to develop a contextually grounded intervention compatible with routine PHC.

This study finding indicate that adolescent SRH service utilization is shaped by interconnected barriers across adolescents, providers, health facilities, and information and referral systems, rather than by a single determinant. This finding is consistent with previous evidence identifying limited information, stigma, confidentiality concerns, provider attitudes, and health-service barriers as overlapping constraints to adolescent SRH care [8,9,30]. This highlight that addressing only adolescents’ knowledge or motivation may be insufficient when services remain inaccessible or unresponsive, while improving facilities alone may have limited impact if adolescents cannot access trusted information or navigate care. Effective interventions therefore need to address both demand-side and supply-side barriers across the pathway to care.

A second key finding was the importance of provider responsiveness and the quality of interpersonal interactions in adolescent SRH care. Stakeholders emphasized that effective adolescent-responsive services require more than technical competence; they also depend on providers’ attitudes, communication, confidentiality, counseling, and ability to deliver respectful and non-judgmental care. This finding is consistent with previous research identifying provider attitudes and competencies as important barriers to adolescent SRH service utilization [8,9,30], as concerns about judgment, privacy, and confidentiality may discourage adolescents from seeking or returning for care. These findings highlight the need to strengthen both technical and interpersonal competencies. Moreover, because a one-time training may be insufficient to sustain changes in provider behavior, the inclusion of mentorship, supportive supervision, refresher learning, and structured feedback in the intervention package emphasizes the importance of continuous reinforcement through routine health-system support mechanisms [26].

A third key finding was the importance of mechanisms that connect adolescents with formal health services while reducing informational, social, and confidentiality-related barriers to care-seeking. Stakeholders identified peer navigation and confidential communication as potentially useful for providing trusted information, addressing sensitive concerns, and helping adolescents navigate services before and after facility visits. This is consistent with evidence from Zambia and Kenya showing that peer-supported approaches can improve adolescents’ access to information and linkage to services [31,32], while digital interventions can provide confidential access to SRH information and support outside conventional face-to-face services [33]. However, information and navigation alone are unlikely to improve service utilization unless they are linked to responsive services and reliable referral pathways. This highlights the importance of integrating demand-side strategies with supply-side improvements in PHC to ensure that adolescents who are reached can successfully access appropriate and acceptable care.

A fourth key finding was that adolescent responsiveness extends beyond provider behavior to the organization and environment of PHC facilities. Stakeholders emphasized privacy, convenient and understandable service processes, respectful treatment, appropriate communication, and a welcoming environment, consistent with evidence linking accessibility, convenience, privacy, and other facility characteristics with young people’s use and satisfaction with SRH services [16,34]. These findings support strengthening adolescent responsiveness within routine PHC rather than relying solely on separate adolescent services, which may be more feasible in resource-constrained settings. However, integration alone does not guarantee equitable access or sustainability; its success depends on facility capacity, resources, supportive management, and continued attention to adolescents’ needs. Future research should therefore assess whether these adaptations can be sustained within routine PHC while improving reach and service utilization without placing undue demands on existing systems.

A fifth key finding was the contribution of HCD and iterative refinement to developing a contextually appropriate intervention. HCD provided a structured process for incorporating the perspectives of adolescents, healthcare providers, facility managers, district officials, and community representatives from barrier identification and prioritization through ideation, prototyping, pilot testing, and refinement. This is consistent with evidence highlighting stakeholder engagement, contextual adaptation, and ownership in adolescent health intervention development [24,26,35]. This might be evidence by showing how stakeholder input can be carried through successive stages of intervention development within routine PHC, rather than being limited to initial needs assessment or idea generation. Pilot testing, supportive supervision, and stakeholder feedback further enabled practical challenges in workflows, roles, and service linkages to be identified and addressed before subsequent implementation [8,36,37]. Thus, the value of HCD in this study lay not only in generating locally relevant solutions but also in iteratively adapting them to the realities of routine PHC delivery. However, contextual fit and stakeholder acceptability do not establish intervention effectiveness; whether the package improves service utilization, health outcomes, equity, or sustainability requires subsequent implementation and effectiveness evaluation.

### Strengths and limitations

This study has several strengths. The structured, iterative HCD process engaged diverse stakeholders, including adolescents, providers, facility managers, health officials, and community representatives, ensuring that the intervention reflected both user and service-delivery perspectives. Pilot testing in routine PHC facilities further strengthened its contextual relevance and practical feasibility.

Several limitations should be considered. The study was conducted in selected PHC facilities in Southern Ethiopia, which may limit transferability to settings with different health-system contexts and service-delivery arrangements. Stakeholder perspectives may also have influenced the prioritization and refinement of intervention components. Importantly, the study focused on intervention development and refinement and did not assess effectiveness; therefore, its effects on ASRH service utilization, health outcomes, equity, and sustainability remain to be established through subsequent evaluation.

## Conclusion

This study successfully co-designed and refined an integrated intervention package to strengthen adolescent sexual and reproductive health services within routine primary healthcare units in Southern Ethiopia using a human-centered design approach. By engaging adolescents, healthcare providers, facility managers, health officials, and community stakeholders throughout the design process, the study translated context-specific challenges into four complementary components: provider and facility manager capacity strengthening, adolescent peer navigation, a confidential SRH call center, and strengthened adolescent-responsive PHC services. The integrated package addresses interconnected barriers across service provision, access, information, trust, and navigation while building on existing PHC structures rather than creating stand-alone services. The study demonstrates the potential of participatory and iterative design to develop a contextually responsive and practically feasible intervention for adolescent SRH in routine PHC settings. Further implementation and effectiveness research is needed to determine its effects on service utilization, equity, health outcomes, and sustainability.

### Policy and practical implications

This study provides compelling evidence that co-designed; HCD interventions can overcome multi-level barriers to ASRH service utilization, enhancing accessibility, acceptability, and engagement. **Policy implications** are clear: national ASRH strategies should formally integrate HCD and participatory co-design principles to ensure interventions are contextually relevant, adolescent-centered, and responsive to real-world challenges. Policymakers should prioritize system-level, multi-component approaches that combine structural improvements such as adolescent-friendly facilities and provider capacity building with demand-side strategies like peer navigation and confidential call centers, thereby maximizing reach and sustainability.

**For program implementers**, the findings underscore the importance of actively engaging adolescents, healthcare providers, facility managers, youth leaders and researchers throughout intervention design and implementation. Iterative prototyping and pilot testing enable alignment with facility workflows and community norms, strengthening feasibility, ownership, and long-term uptake. The low-cost, scalable prototypes developed—provider capacity building, peer navigators, SRH call centers, and adolescent-friendly service environments—offer a practical blueprint for replication in low-resource settings.

**Scalability and sustainability** can be achieved by integrating these interventions into routine primary healthcare services, phasing implementation to optimize resources, and evaluating cost-effectiveness and long-term impact. Collectively, these findings provide actionable guidance for policymakers, program implementers, and researchers seeking to design and scale effective, adolescent-centered SRH services.

## Supporting information

S1 Appendix Detailed facilitator guides and operational materials for the human-centered design co-design workshops. Facilitator guides and operational materials, including workshop agendas, activity instructions, guiding questions, and facilitation prompts used during the co-design workshops.
(DOCX)

S2 Appendix Problem prioritization criteria and weighted multi-criteria decision analysis framework. Complete multi-criteria decision analysis (MCDA) framework, including criterion definitions, scoring anchors, assigned weights, and the scoring and ranking matrix used to prioritize identified problems.
(XLSX)

S3 Appendix Consolidated list and classification of identified barriers. Consolidated list and classification of 57 unique barriers identified during the formative assessment and human-centered design process, organized into relevant thematic domains.
(DOCX)

S4 Appendix Detailed description of the final integrated intervention package. Description of the four components of the final integrated intervention package, including their objectives, key activities, delivery mechanisms, and implementation responsibilities.
(DOCX)

S5 Appendix Overview of the human-centered design co-design process for developing the adolescent-responsive primary healthcare intervention in Southern Ethiopia. Detailed overview of the HCD phases, activities, and stakeholder engagement processes undertaken to develop the intervention.
(DOCX)
