# Supplementary material for: Co-designing an integrated intervention package to strengthen adolescent sexual and reproductive health services in primary healthcare units in Southern Ethiopia: A human-centered design approach
Source: PLOS Glob Public Health. 2026 Nov 11;6(11):e0007554. doi: 10.1371/journal.pgph.0007554 (PMC13754124; doi:10.1371/journal.pgph.0007554)
Supplement: Supplementary file 1 [file pgph.0007554.s001.docx]

**Project Title:** Designing and Pilot-Testing an Integrated Intervention Package to Strengthen Primary Health Care Services for Improved Adolescent Sexual and Reproductive Health Outcomes in Southern Ethiopia
**Study Approach:** Human-Centered Design (HCD)-guided participatory intervention co-design and pilot testing
**Study Period:** May–October 2025

**Study Setting:** Primary healthcare (PHC) facilities and community engagement sites in Southern Ethiopia

## Part A. Methodological Framework and Co-Design Process

### 1. Overview of the Human-Centered Design Approach

#### 1.1. Rationale and Theoretical Framework

This supplementary file describes the methodological framework and operational procedures used to co-design an integrated Adolescent-Responsive Primary Healthcare (ARPHC) intervention package for adolescent sexual and reproductive health (ASRH) services in Southern Ethiopia. Barriers to adolescent-responsive service delivery in PHC settings include provider-associated stigma, inadequate auditory and visual privacy, fragmented referral linkages, and limited youth-friendly service arrangements.

The intervention was developed using HCD approach that places adolescents’ lived experiences, needs, and preferences alongside the operational realities of healthcare providers and the health system. The process emphasized empathic inquiry, collaborative problem definition, participatory solution generation, low-fidelity prototyping, field testing, and iterative refinement within routine PHC settings.

#### 1.2. Stakeholder Composition and Selection Criteria

The co-design process engaged five stakeholder groups to incorporate perspectives from service users, frontline service providers, health system administrators, and the wider community. Stakeholders were included based on their roles and relevance to the design and implementation of adolescent-responsive PHC services.

1. Adolescents and young people (service users): Contributed experiences of service access and utilization, unmet SRH needs, privacy and confidentiality concerns, perceived stigma, and preferences for service delivery.
2. Healthcare providers (frontline implementers): Provided clinical and operational perspectives on adolescent-responsive care, routine workflows, workload, and implementation barriers.
3. Facility managers (health center leadership): Contributed insights into staffing, resource allocation, service organization, supervision, and integration of the intervention into facility operations.
4. District health officials (health system administration): Provided perspectives on health system coordination, local priorities, resource and logistics management, and potential for scale-up.
5. Community representatives (local context experts): Contributed insights into sociocultural norms, community expectations, parental perspectives, and approaches to community engagement and sensitization.

#### 1.3. Five-Phase HCD Sequence and Overall Design Logic

Intervention development followed a five-phase HCD framework comprising Empathize, Define, Ideate, Prototype, and Test and Refine. The process involved structured workshops and complementary field activities, with findings from each phase informing subsequent development and refinement of the ARPHC intervention package.

- **Empathize** (Workshop 1): Explored adolescent and provider experiences, validated service barriers, and mapped service journeys.
- **Define** (Workshop 2): Synthesized findings through affinity mapping, developed problem statements, prioritized barriers using multi-criteria decision analysis (MCDA), and formulated How Might We (HMW) questions.
- **Ideate (**Workshop 3): Generated and clustered potential solutions and prioritized concepts using a Feasibility–Impact Matrix.
- **Prototype** (Workshop 4): Translated prioritized concepts into low-fidelity intervention components, mapped service workflows, and conducted role-play simulations.
- **Test and Refine** (subsequent workshops and field activities): Conducted implementer training, piloted the intervention in four health centers, undertook supportive supervision, and incorporated stakeholder feedback to refine and consolidate the final intervention package.

### 2. Phase 1: Empathize Phase Methodology

#### 2.1. Purpose, Objectives, and Review of Formative Findings

The Empathize phase aimed to develop a context-specific understanding of adolescents’ needs, service experiences, and barriers to accessing ASRH services in PHC facilities. Workshop 1 initiated the phase by reviewing synthesized findings from preceding formative research and assessing their relevance to the local service-delivery context.

The specific objectives were to:

- Review formative research findings on ASRH service access and utilization.
- Explore the experiences and expectations of adolescents, healthcare providers, and local health leaders.
- Validate and contextualize identified barriers across PHC settings.
- Establish a consolidated understanding of user and provider challenges to inform problem definition.

#### 2.2. Qualitative Data Collection: Empathy Mapping and Journey Mapping

Participatory qualitative exercises were conducted in mixed and stakeholder-specific small groups through three activities.

- **E1. Orientation and Experience Sharing** (45–60 minutes): Introduced HCD principles, established a respectful environment for participation, and invited stakeholders to share experiences of ASRH services.
- **E2. Empathy Mapping** (60–90 minutes): Explored adolescent service-user experiences across six dimensions: Says (explicit statements), Thinks (concerns and perceptions), Does (care-seeking actions), Feels (emotions), Pain Points (fears, embarrassment, and delays), and Needs (requirements for responsive care).
- **E3. Journey Mapping** (90–120 minutes): Mapped the service-seeking pathway across ten stages: (1) trigger or need for care; (2) information seeking; (3) decision to seek care; (4) facility or provider selection; (5) facility arrival; (6) registration or reception; (7) provider interaction; (8) receipt of ASRH care; (9) referral or follow-up; and (10) post-service re-entry into the community.

#### 2.3. Outputs and Transition to Problem Definition

The Empathize phase generated validated user-experience narratives, empathy maps, service journey maps, and a consolidated list of 57 distinct barriers to ASRH service access and delivery. These outputs informed the synthesis and prioritization activities undertaken during the Define phase.

### 3. Phase 2: Define Phase Methodology

#### 3.1. Affinity Clustering and Problem Framing

Workshop 2 translated findings from the Empathize phase into structured, user-centered problem statements through three activities.

- **D1. Review and Synthesis** (45–60 minutes): Stakeholders reviewed and validated the 57 identified barriers for relevance to adolescents’ experiences and routine PHC settings.
- **D2. Affinity Mapping** (60–90 minutes): Participants grouped related barriers into thematic clusters using sticky notes and visual wall boards to identify common patterns and interconnected challenges.
- **D3. Problem Definition** (60–90 minutes): Thematic findings were translated into user-centered problem statements using a standardized structure: “[User group] experiences [problem] because of [underlying barrier or context], resulting in [negative consequence].”

These activities established a shared understanding of the challenges affecting adolescent-responsive SRH services and provided the foundation for subsequent prioritization and design-question formulation.

#### 3.2. Multi-Criteria Decision Analysis Prioritization

**Activity D4: MCDA Prioritization (90–120 minutes)**

Stakeholders used a structured MCDA process to prioritize identified barriers according to four criteria: public health importance and severity, potential impact on adolescent health outcomes, feasibility within routine PHC settings, and alignment with local health system priorities. The process narrowed the initial 57 barriers to 19 priority problems, which informed the subsequent formulation of design questions. Detailed prioritization results are provided in the accompanying supplementary materials.

#### 3.3. Reframing Priority Problems into “How Might We” Questions

**Activity D5: HMW Question Formulation (60–90 minutes)**

Stakeholders collaboratively reframed the 19 priority problems into 10 open-ended HMW questions, translating identified challenges into actionable design opportunities. The questions reflected adolescents’ needs and the practical realities of routine PHC settings while encouraging creative, contextually appropriate solutions. They subsequently guided ideation, solution prioritization, and prototype development to improve adolescents’ access to and utilization of quality SRH services.

### 4. Phase 3: Ideate Phase Methodology

#### 4.1. Solution Generation and Brainstorming

Workshop 3 addressed the 10 HMW questions to generate a broad range of potential intervention solutions through three activities.

- I1. HMW Orientation (30–45 minutes): Re-established the design challenges and boundaries for solution generation.
- I2. Individual Brainstorming (30–45 minutes): Participants independently recorded ideas on sticky notes to encourage independent contributions and reduce peer-conformity and authority effects.
- I3. Group Brainstorming (60–90 minutes): Multistakeholder groups expanded, combined, and modified ideas using a deferred-judgment approach to encourage creative exploration before solution evaluation.

#### 4.2. Solution Clustering and Feasibility–Impact Assessment

The brainstorming activities generated 140 initial intervention ideas. These were reviewed, consolidated, and assessed through two activities.

- I4. Idea Presentation and Clustering (60–90 minutes): Participants removed exact duplicates and grouped related ideas into consolidated intervention concepts while preserving distinct ideas.
- I5. Feasibility–Impact Matrix Assessment (60–90 minutes): Participants assessed each concept according to its potential impact and implementation feasibility and positioned the concepts on a 2 × 2 Feasibility–Impact Matrix.

| **Implementation feasibility** | **Low potential impact** | **High potential impact** |
| --- | --- | --- |
| High | Secondary priority, resource permitting | Top priority for prototyping |
| Low | Not prioritized for the current package | Consider for contextual adaptation |

This assessment prioritized concepts with high potential impact on ASRH service quality and high feasibility within routine PHC settings. Concepts with high potential impact but lower feasibility could be retained for further consideration where contextual adaptation might improve implementation feasibility.

### 5. Phase 4: Prototype Phase Methodology

#### 5.1. Translation of Concepts into Low-Fidelity Intervention Components

Workshop 4 translated prioritized intervention concepts into tangible, low-fidelity prototypes through Activities P1–P2 (approximately 120 minutes). Prototype formats included:

- Service-flow diagrams and workflows: Proposed arrangements for clinic reception, triage, and consultation pathways.
- Role descriptions and job aids: Standard operating procedures and practical guidance for healthcare providers and peer navigators.
- Information, education, and communication (IEC) tools: Youth-friendly counseling cards and digital referral flowcharts.
- SRH call-center protocols: Confidential telephone and SMS counseling workflows.
- Facility-readiness checklists: Assessment of auditory and visual privacy in consultation rooms.

#### 5.2. Service-Flow Mapping, Role-Play, and Usability Review

Prototype components were reviewed and refined through the following activities.

- P3. Prototype Walkthroughs (60–90 minutes): Conducted step-by-step walkthroughs of user and provider interaction pathways to examine how the proposed components would function in practice.
- P4. Role-Play and Simulation (60–120 minutes): Rehearsed realistic service scenarios using scenario cards and adolescent, provider, and peer-navigator roles.
- P5–P6. Prototype Review and Refinement (60–90 minutes): Used simulation debriefs and stakeholder feedback to revise tools and prepare the intervention components for pilot implementation.

### 6. Phase 5: Test and Refine Phase Methodology

#### 6.1. Implementer Training

Before field testing, a three-day training program was conducted for healthcare providers and facility managers from participating health facilities. The training covered ASRH principles, the rationale for the HCD co-design process, the components of the co-designed intervention package, peer navigation, adolescent-responsive communication, privacy and confidentiality protection, service-flow simulation, and strategies for addressing potential implementation challenges. Following this initial training, three participants were selected as trainers to conduct separate three-day training for adolescent and youth peer navigators. The second training focused on the roles and responsibilities of peer navigators, effective and nonjudgmental communication with adolescents and young people, the use of intervention tools, protection of privacy and confidentiality, identification of adolescents’ support needs, and appropriate referral and linkage to health services. It also addressed practical approaches to supporting adolescents and young people in accessing and navigating ASRH services within participating health facilities.

#### 6.2. Pilot Implementation in Four Primary Healthcare Facilities

The pilot-ready intervention package was implemented under routine service-delivery conditions in four selected health centers in Southern Ethiopia over an eight-week period. The pilot provided an opportunity to examine the use of intervention components within existing service workflows and identify practical implementation challenges and areas requiring adaptation.

#### 6.3. Supportive Supervision and Participatory Feedback

Supportive supervision visits: Research supervisors conducted periodic, non-punitive visits using structured checklists to assess intervention fidelity, workflow integration, tool usability, privacy and confidentiality, and workload implications. Supervisors provided constructive feedback and documented implementation challenges and opportunities for improvement.

Participatory feedback workshop: Adolescents, healthcare providers, facility managers, and district health leaders reviewed pilot implementation records and supervision findings, discussed user experiences, and identified implementation challenges. Participants collaboratively prioritized adaptations to improve the intervention’s acceptability, feasibility, usability, and contextual relevance.

#### 6.4. Iterative Refinement and Final Intervention Consolidation

Findings from the pilot, supportive supervision, and stakeholder feedback were synthesized using a component-refinement framework comprising six actions: Retain, Modify, Add, Remove, Clarify, and Adapt. This process guided evidence-informed adjustments to intervention components, delivery procedures, and implementation guidance. The process culminated in consolidation of the final ARPHC intervention package, as described in the main manuscript.

**PART B. FACILITATOR GUIDES AND OPERATIONAL TEMPLATES**

**1. Facilitation Standards and Workshop Management**

**1.1. Facilitation team roles and responsibilities**

| **Team member** | **What the person does** |
| --- | --- |
| Lead facilitator | Prepares the session plan, introduces objectives, explains activities, gives instructions, asks probing questions, manages time, facilitates plenary discussions, resolves misunderstandings, and summarizes decisions. |
| Co-facilitator | Prepares materials, organizes small groups, repeats or clarifies instructions, encourages quieter participants, monitors group dynamics, assists with templates, and alerts the lead facilitator to emerging issues. |
| Rapporteur/note-taker | Records participant contributions, key quotations where appropriate, disagreements, scoring results, decisions, and reasons for decisions. Collects completed worksheets and organizes visual documentation. |
| Design team | Provides relevant formative evidence, supports interpretation of findings, helps participants develop and document concepts, and maintains consistency across the five HCD phases. |
| Workshop participants | Share experiences, identify barriers, generate and assess ideas, develop prototypes, provide feedback, and contribute to decisions about intervention refinement. |

**1.2. Standard facilitator procedure**

The lead facilitator should follow these steps during each workshop.

1. Before the session: Review the objectives and agenda, prepare materials and templates, arrange the venue, confirm participant groups, and agree on the roles of the facilitation team.
2. At the beginning: Welcome participants, explain the purpose of the session, review the agenda and ground rules, and clarify how the outputs will inform intervention development.
3. During activities: Explain each task, demonstrate the process when necessary, ask open-ended questions, encourage balanced participation, monitor time, and ensure that participants understand what they are expected to produce.
4. During group reporting: Invite each group to present its findings, ask clarifying questions, identify similarities and differences, and record areas of agreement and disagreement.
5. During decision-making: Explain the relevant decision criteria, give participants an opportunity to discuss alternatives, document the rationale for decisions, and avoid presenting facilitator preferences as stakeholder consensus.
6. At the end: Summarize the outputs, check their accuracy with participants, identify unresolved issues, explain the next steps, and ensure that completed worksheets and other records are collected.

**1.3. Ground rules, inclusivity, and power dynamics**

The facilitator should:

- Ask participants to agree on ground rules at the beginning of the workshop.
- Explain that all perspectives are valuable, regardless of age, professional position, or administrative rank.
- Invite participants to write ideas individually before discussing them in groups.
- Use separate adolescent or youth group discussions when appropriate to reduce the influence of adults or senior professionals.
- Invite contributions from quieter participants without forcing anyone to speak.
- Use anonymous voting when participants may feel uncomfortable expressing disagreement publicly.
- Redirect dominant speakers respectfully and provide opportunities for alternative views.
- Avoid requiring adolescents to disclose personal sexual or reproductive health experiences.
- Remind participants to respect the privacy of information shared, while explaining that confidentiality among workshop participants cannot be guaranteed absolutely.

**1.4. Venue preparation and documentation**

Workshop venues were arranged to support plenary discussions, small-group activities, collaborative mapping, and presentation of outputs.

Preparation included arranging seating to facilitate interaction, establishing breakout areas, preparing wall or board space for displaying workshop outputs, and organizing the materials required for each activity.

Materials checklist

- Flipcharts and flipchart stands
- Multicolored sticky notes
- Thick markers and pens
- Adhesive tape
- Printed empathy maps and journey-mapping templates
- Problem-framing and weighted multi-criteria decision analysis (MCDA) matrices
- Feasibility–Impact matrices
- Prototype worksheets and scenario cards
- Supervision and refinement templates
- Laptops or other approved documentation equipment, where required

Workshop notes, completed templates, photographs of group outputs, and decision records were organized to support traceability across the five HCD phases.

**2. Step-by-Step Workshop Facilitation Guides**

**2.1. Workshop 1: Empathize Phase**

Primary objective: To explore adolescent and provider experiences, validate formative research findings, identify unmet needs, and map the adolescent ASRH service journey.

**Activity E1. Participant orientation and experience sharing (45–60 minutes)**

Materials: Agenda, presentation slides, flipcharts, markers, and discussion prompts.

Facilitator procedure

1. Welcome participants and explain the purpose of the workshop.
2. Introduce the HCD approach and explain why stakeholder experiences are important for designing adolescent-responsive ASRH services.
3. Present the agenda and explain the expected outputs.
4. Review the ground rules and invite participants to suggest additional rules.
5. Ask participants to reflect individually on their experiences of accessing, delivering, or managing ASRH services.
6. Invite participants to share their observations in pairs or small groups before opening the plenary discussion.
7. Ask open-ended questions to explore barriers, enabling factors, and unmet needs.
8. Record important observations on flipcharts or sticky notes using participants' own words where appropriate.
9. Summarize the major themes and ask participants whether the summary accurately reflects their contributions.

Guiding questions

- What makes it easy or difficult for adolescents to seek ASRH services?
- What challenges do providers face when serving adolescents?
- What experiences influence adolescents' trust in healthcare services?
- What changes would make services more accessible and acceptable?

Expected output: A documented set of stakeholder experiences, initial service barriers, and unmet needs.

**Activity E2. Empathy mapping (60–90 minutes)**

Materials: Large empathy-map templates, sticky notes, markers, and available formative research summaries.

Facilitator procedure

1. Divide participants into small groups, ensuring appropriate representation of stakeholder perspectives.
2. Distribute one empathy-map template to each group.
3. Explain the six domains: Says, Thinks, Does, Feels, Pain Points, and Needs.
4. Ask groups to select the user group or persona they will explore, such as adolescent service users or healthcare providers.
5. Instruct participants to write one observation or insight per sticky note and place it in the appropriate domain.
6. Encourage participants to distinguish documented evidence from assumptions or interpretations.
7. Ask probing questions when groups make broad statements without explaining the underlying experience.
8. Invite groups to identify recurring pain points, unmet needs, and opportunities for improvement.
9. Ask each group to present its empathy map.
10. Facilitate comparison across groups and document shared findings and important differences.

Guiding questions

- What might an adolescent be worried about but hesitate to say?
- How might fear of judgment influence help-seeking?
- What does the adolescent experience when interacting with reception staff or providers?
- What needs remain unmet even after an adolescent reaches a facility?

Expected output: Completed empathy maps with documented pain points, needs, and opportunities for intervention.

**Activity E3. Service journey mapping (90–120 minutes)**

Materials: Journey-map templates, sticky notes, markers, and formative research findings.

Facilitator procedure

1. Explain that journey mapping examines the sequence of experiences through which an adolescent seeks and receives services.
2. Ask groups to identify the journey stages used in the study, from recognizing a need for care to receiving services and any subsequent referral or follow-up.
3. Draw or display the journey stages on a large template.
4. Ask participants to describe the adolescent's actions and decisions at each stage.
5. Invite participants to identify interactions with providers, reception staff, peer navigators, and other relevant service personnel.
6. Ask participants to describe emotional experiences and barriers at each stage.
7. Mark points where adolescents may delay, abandon, or experience difficulty accessing care.
8. Ask groups to identify opportunities for improving the journey at each point.
9. Invite groups to present their maps and compare findings.
10. Consolidate recurring barriers and opportunities, preserving important differences between stakeholder groups.

Guiding questions

- At which stage is an adolescent most likely to experience difficulty?
- What happens if an adolescent is concerned about confidentiality?
- Where do delays, unnecessary referrals, or communication failures occur?
- Which changes could improve the service experience?

Expected output: Completed service journey maps and a consolidated list of barriers and opportunities.

**2.2. Workshop 2: Define Phase**

Primary objective: To synthesize the Empathize findings, formulate user-centered problem statements, prioritize problems through weighted MCDA, and develop HMW questions.

**Activity D1–D2. Findings review and affinity mapping (105–150 minutes)**

Materials: Barrier statements, sticky notes, markers, wall space, and affinity-mapping templates.

Facilitator procedure

1. Present the findings from Workshop 1 and explain how the barriers were documented.
2. Display the identified barrier statements so participants can review them.
3. Clarify that the purpose is to organize and interpret the findings rather than immediately select solutions.
4. Ask participants to read the statements and identify barriers that appear conceptually related.
5. Invite participants to move related statements into clusters without assigning cluster names initially.
6. Encourage discussion when participants disagree about where a statement belongs.
7. Ask groups to identify the central theme represented by each cluster.
8. Write a concise label for each cluster and review whether it accurately represents the grouped barriers.
9. Identify overlapping clusters and discuss whether they should be merged or retained separately.
10. Present the consolidated clusters in plenary and ask participants to validate the results.
11. Record the final cluster names and any unresolved differences.

Expected output: A documented set of thematic barrier clusters.

**Activity D3. Problem definition (60–90 minutes)**

Materials: Problem-framing worksheets and consolidated barrier clusters.

Facilitator procedure

1. Explain the difference between a barrier, an underlying cause, a problem, and a consequence.
2. Assign each group one or more thematic clusters.
3. Ask participants to identify the affected user group and the service context.
4. Ask participants to distinguish the underlying cause from the observable problem.
5. Guide participants to formulate problem statements using this structure:

[User group] experiences [problem] because of [underlying cause], resulting in [consequence].

6. Ask groups to check whether each statement is specific, evidence-informed, and focused on an issue that could be addressed through service or system improvements.
7. Encourage participants to avoid embedding a predetermined solution in the problem statement.
8. Invite groups to present their statements.
9. Facilitate revision where statements are vague, overlapping, or overly broad.
10. Record the agreed statements and assign identification codes.

Expected output: Structured, documented problem statements linked to the formative findings.

**Activity D4. Weighted MCDA prioritization (90–120 minutes)**

Materials: MCDA scoring matrix, agreed criteria and weights, scoring instructions, and calculators or spreadsheets.

Facilitator procedure

1. Explain why prioritization is needed when many problems have been identified but time and resources are limited.
2. Present the candidate problem statements and confirm that participants understand each one.
3. Review the prioritization criteria and explain the meaning of each criterion.
4. Present the agreed weights and explain how the weights influence the final scores.
5. Explain the scoring scale and provide a brief example using a noncontroversial practice item.
6. Ask participants to score each problem against each criterion, following the actual scoring method used in the study.
7. Where appropriate, allow independent scoring before discussing differences.
8. Facilitate discussion of substantially different scores and document the reasons for disagreement.
9. Calculate the weighted score for each problem using the agreed formula.
10. Rank the problems according to their weighted totals.
11. Present the rankings to participants and invite them to check for calculation errors or unintended consequences.
12. Document the final ranking, any agreed adjustments, and the rationale for decisions.

The facilitator should not change weights or scores simply to obtain a preferred ranking. Any changes must follow the agreed decision procedure and be documented.

Expected output: A completed MCDA matrix and a transparent, documented ranking of priority problems.

**Activity D5. HMW question formulation (60–90 minutes)**

Materials: Prioritized problem statements and HMW worksheets.

Facilitator procedure

1. Display the prioritized problems.
2. Explain that an HMW question should frame a design opportunity without prescribing a particular solution.
3. Demonstrate how to convert one problem statement into an open-ended question.
4. Ask small groups to draft HMW questions for their assigned problems.
5. Review whether each question identifies a meaningful user need and leaves room for multiple possible solutions.
6. Ask groups to combine questions that address the same underlying design challenge.
7. Check that the questions do not imply that a particular intervention is already the preferred answer.
8. Invite participants to review and refine the questions.
9. Record the final HMW questions and link them to the corresponding prioritized problems.

Expected output: A documented set of HMW design questions linked to priority problems.

**2.3. Workshop 3: Ideate Phase**

Primary objective: To generate a broad range of ideas, consolidate them into solution concepts, and prioritize concepts for prototyping.

**Activity I1–I2. HMW review and individual brainstorming (60–90 minutes)**

Materials: HMW cards, sticky notes, markers, and idea-generation worksheets.

Facilitator procedure

1. Present the agreed HMW questions and explain the problem each question addresses.
2. Explain brainstorming ground rules: defer judgment, encourage variety, build on others' ideas, and consider unconventional approaches.
3. Ask participants to work independently for an initial period.
4. Instruct each participant to write one idea per sticky note, using concise descriptions.
5. Encourage participants to consider service delivery, provider practices, peer support, communication, referral pathways, and organizational arrangements where relevant.
6. Remind participants that the goal at this stage is to generate alternatives rather than evaluate feasibility.
7. Collect and display the ideas, ensuring that their links to the relevant HMW questions are retained.

Expected output: A documented pool of individual solution ideas.

**Activity I3. Group brainstorming and co-creation (60–90 minutes)**

Materials: Individual ideas, group worksheets, flipcharts, and markers.

Facilitator procedure

1. Organize participants into small groups.
2. Ask groups to review the ideas generated individually.
3. Invite participants to explain ideas that are unclear.
4. Ask groups to identify opportunities to combine, extend, or improve ideas.
5. Encourage participants to consider different stakeholder perspectives and alternative delivery approaches.
6. Record new ideas separately so they are not confused with the original submissions.
7. Ask groups to identify ideas that address the same problem through different mechanisms.
8. Document the expanded pool while preserving the original ideas for traceability.

Expected output: An expanded and documented pool of solution ideas.

**Activity I4–I5. Clustering and Feasibility–Impact assessment (120–180 minutes)**

Materials: Idea cards, clustering worksheets, Feasibility–Impact Matrix, and rating instructions.

Facilitator procedure

1. Display the ideas generated during the brainstorming activities.
2. Explain that similar ideas will be grouped into broader concepts, while distinct ideas should be preserved.
3. Ask participants to identify ideas addressing similar needs or using similar mechanisms.
4. Invite participants to move related ideas into clusters.
5. Ask groups to label each cluster with a short description of the common solution approach.
6. Review possible duplicates and ask participants whether they are genuinely equivalent or represent different approaches.
7. Develop a consolidated concept statement for each cluster, describing the proposed solution, target users, and intended purpose.
8. Explain the impact and feasibility criteria and the rating scales actually used.
9. Ask participants to assess each concept against these criteria.
10. Record the ratings and explain any differences that emerge during discussion.
11. Plot the concepts on the 2 × 2 matrix according to the agreed assessment method.
12. Invite participants to review the positioning and identify concepts with high impact and high feasibility.
13. Discuss promising concepts outside the high-impact/high-feasibility quadrant, including whether they could be adapted or require additional resources.
14. Facilitate the selection of concepts for prototyping using the agreed decision process.
15. Document the selected concepts, concepts retained for future consideration, and reasons for excluding or deferring other concepts.

Expected output: A consolidated pool of solution concepts, a completed Feasibility–Impact Matrix, and a documented shortlist of concepts selected for prototyping.

**2.4. Workshop 4: Prototype Phase**

Primary objective: To translate prioritized concepts into low-fidelity prototypes, map operational workflows, and identify problems through structured walkthroughs and role-play.

**Activity P1–P2. Concept review and prototype development (120–165 minutes)**

Materials: Selected concepts, prototype worksheets, paper, markers, flowchart templates, and relevant existing service materials.

Facilitator procedure

1. Present the concepts selected during the Ideate phase.
2. Ask participants to confirm the intended purpose and target users of each concept.
3. Divide participants into groups and assign each group a concept or related set of concepts.
4. Explain that a low-fidelity prototype is a preliminary representation used to examine how an intervention might work; it need not be a finished product.
5. Ask each group to describe the intended user, the problem addressed, the proposed activities, and the expected service pathway.
6. Provide relevant templates and ask groups to produce a preliminary representation, such as a flowchart, job aid, counseling card, or peer navigation procedure.
7. Ask groups to identify the staff, materials, space, and organizational arrangements needed.
8. Review each prototype for clarity, usability, and consistency with the agreed intervention concept.
9. Record unresolved questions for testing or subsequent refinement.

Expected output: Preliminary intervention prototypes and documented operational requirements.

**Activity P3–P4. Walkthroughs and role-play simulations (120–210 minutes)**

Materials: Prototype materials, scenario cards, observation sheets, and feedback forms.

Facilitator procedure

1. Explain the purpose of the simulation and clarify that it is intended to test the proposed process, not evaluate individual performance.
2. Select scenarios reflecting realistic service situations.
3. Assign roles appropriate to the scenario, such as adolescent service user, healthcare provider, or peer navigator.
4. Ask participants to use the prototype while acting out the scenario.
5. Ask observers to document steps that are unclear, difficult, delayed, or potentially inconsistent with confidentiality requirements.
6. Avoid interrupting the simulation unless clarification or safety requires it.
7. After each simulation, invite the participants who played the roles to describe their experiences.
8. Ask observers to present their recorded findings.
9. Identify where the prototype supported the service process and where it created difficulties.
10. Record each problem, the supporting observation, and a proposed modification.

Expected output: Simulation records, documented usability and workflow problems, and proposed modifications.

**Activity P5–P6. Debriefing and prototype refinement (120–150 minutes)**

Materials: Prototype worksheets, simulation debrief forms, and revision materials.

Facilitator procedure

1. Summarize the problems identified during the simulations.
2. Ask participants to distinguish essential changes from minor improvements.
3. Discuss the implications of each proposed change for users, providers, workflow, resources, and confidentiality.
4. Ask groups to revise their prototypes.
5. Compare revised versions with the original concepts to ensure that the intended function is retained.
6. Record the changes made and the reasons for them.
7. Identify unresolved issues that require clarification or testing during field implementation.
8. Review the revised prototypes with the wider group.
9. Document the components that are ready for field testing and any remaining preparation requirements.

Expected output: Revised prototypes, documented workflow changes, and a record of outstanding issues to be addressed before or during field testing.

**2.5. Training and Preparation for Field Testing**

Training was conducted in two stages, reflecting the different responsibilities of healthcare providers and facility managers, and adolescent and youth peer navigators.

**Stage 1. Training for healthcare providers and facility managers**

Duration: Three days.

Facilitator/trainer procedure

1. Explain the objectives of the training and the intended role of each participant in implementing the intervention.
2. Introduce ASRH principles and the rationale for the HCD co-design process.
3. Review the intervention components and demonstrate how they relate to the identified service barriers.
4. Explain the peer navigation approach and the respective responsibilities of health facility staff and peer navigators.
5. Demonstrate adolescent-responsive communication and discuss how to protect privacy and confidentiality.
6. Guide participants through service-flow simulations.
7. Present scenarios involving possible implementation challenges and ask participants to discuss appropriate responses.
8. Provide opportunities to practice using the intervention materials and tools.
9. Ask facility teams to identify staffing, space, workflow, and resource requirements for implementation.
10. Review outstanding preparation needs and document the agreed actions.

Expected output: Healthcare providers and facility managers oriented to the intervention and prepared for their assigned implementation responsibilities.

**Stage 2. Training for adolescent and youth Peer navigators**

Duration: Three days.

Trainers: Three participants selected following the initial training.

Trainer procedure

1. Introduce the purpose of peer navigation and explain the role of peer navigators within the co-designed intervention.
2. Clarify the boundaries of the peer navigator role, including the distinction between providing peer support and providing clinical assessment or treatment.
3. Explain how peer navigators can support adolescents in accessing and navigating ASRH services.
4. Demonstrate respectful, nonjudgmental communication and discuss how to respond to adolescents' questions and concerns.
5. Explain privacy and confidentiality responsibilities and the appropriate handling of sensitive information.
6. Demonstrate how to use the relevant intervention tools.
7. Explain the circumstances in which adolescents should be linked or referred to healthcare providers, following the agreed referral procedures.
8. Use realistic scenarios to practice communication, navigation, and referral.
9. Invite trainees to discuss difficulties and ask questions.
10. Review the key responsibilities and document any additional support or clarification needed before field testing.

Expected output: Adolescent and youth Peer navigators oriented to their roles and prepared for the activities assigned to them.

**2.6. Workshop 5: Test and Refine Phase**

Primary objective: To review field-testing experiences, examine implementation evidence, and make documented decisions about the final intervention package.

**Activity T1–T3. Field-testing and supervision review (90–120 minutes)**

Materials: Field-testing summaries, available supervision records, feedback summaries, and the HCD Decision Log.

Facilitator procedure

1. Explain the purpose of the review and clarify that decisions should be based on documented field experiences and stakeholder feedback.
2. Present a summary of the field-testing process in the four participating healthcare facilities.
3. Explain the available evidence, including its source and any limitations.
4. Invite participants to identify what worked as intended and what proved difficult to implement.
5. Ask participants to distinguish problems affecting the intervention itself from wider contextual or organizational barriers.
6. Record each issue and the evidence supporting it.
7. Ask participants to identify whether further clarification is needed before a decision can be made.
8. Summarize the principal implementation strengths and challenges.

Expected output: A consolidated record of field-testing experiences, implementation strengths, and outstanding challenges.

**Activity T4–T5. Stakeholder assessment (120–150 minutes)**

Materials: Feedback templates, component summaries, and feasibility and usability worksheets.

Facilitator procedure

1. Divide participants into groups appropriate to the feedback task.
2. Present each intervention component and explain its intended purpose.
3. Ask participants to assess the component's acceptability, usability, feasibility, and fit with routine services, as applicable.
4. Encourage adolescents and other service users to discuss their experiences without requiring personal disclosures.
5. Ask healthcare providers and managers to identify workload, workflow, staffing, space, and resource implications.
6. Invite peer navigators to discuss role clarity, communication, and referral processes.
7. Record positive experiences, concerns, and suggestions separately.
8. Ask groups to provide evidence or examples supporting their observations.
9. Compare findings across stakeholder groups and facilities.
10. Document areas of agreement and disagreement without assuming that all participants share the same views.

Expected output: Documented stakeholder feedback on the intervention components and their implementation requirements.

**Activity T6–T7. Component refinement and finalization (120–150 minutes)**

Materials: Component Refinement Matrix, HCD Decision Log, and revised prototype materials.

Facilitator procedure

1. Present the consolidated issues and feedback from the field-testing review.
2. Review each intervention component in turn.
3. Ask participants to identify the evidence supporting retention, modification, addition, removal, clarification, or adaptation of the component.
4. Discuss the implications of each proposed decision for adolescent users, providers, service delivery, resources, and implementation.
5. Record the proposed decision and its rationale in the Component Refinement Matrix.
6. Facilitate discussion until the decision process specified for the workshop has been followed.
7. Document any disagreement or unresolved issue.
8. Ask the design team to update the intervention materials and workflows in accordance with the decisions made.
9. Present the revised package for stakeholder verification.
10. Check that the documented modifications are reflected in the final version.
11. Complete the HCD Decision Log, identifying the issue, evidence source, decision, resulting modification, and implementation status.
12. Summarize the final package and any outstanding issues.

Expected output: A completed Component Refinement Matrix, an updated HCD Decision Log, and a documented final intervention package.

**3. Standardized Worksheets and Operational Templates**

The templates below support consistent documentation across the HCD process. Facilitators should explain how to complete each template, check entries for clarity, and collect the completed records at the end of the relevant activity.

**3.1. Empathy and journey mapping templates (K1)**

**Template K1a. Empathy mapping worksheet**

| **Persona/group** | **Says** | **Thinks** | **Does** | **Feels** | **Pain points** | **Unmet needs** |
| --- | --- | --- | --- | --- | --- | --- |
| Adolescent service user |  |  |  |  |  |  |
| Healthcare provider |  |  |  |  |  |  |

Facilitator instructions: Ask participants to focus on the selected persona, write one observation per note, distinguish evidence from assumptions, and explain the basis for important interpretations. Review the completed map with the group and clarify any ambiguous entries.

**Template K1b. Service journey mapping worksheet**

| **Journey stage** | **User actions** | **Provider interactions** | **Emotional experience** | **Identified barriers** | **Service opportunities** |
| --- | --- | --- | --- | --- | --- |
| Stage 1 |  |  |  |  |  |
| Stage 2 |  |  |  |  |  |
| Stage 3 |  |  |  |  |  |
| Stage 4 |  |  |  |  |  |
| Stage 5 |  |  |  |  |  |
| Stage 6 |  |  |  |  |  |
| Stage 7 |  |  |  |  |  |
| Stage 8 |  |  |  |  |  |
| Stage 9 |  |  |  |  |  |
| Stage 10 |  |  |  |  |  |

Facilitator instructions: Ask participants to use the journey stages actually established during the study. At each stage, record the user's actions, interactions, emotional experiences, barriers, and possible improvements. If 10 stages were used, enter their actual names in the template.

**3.2. Problem framing and weighted MCDA templates (K2)**

**Template K2a. Structured problem-framing worksheet**

| **Issue ID** | **Affected group** | **Service context** | **Underlying cause** | **Problem statement** | **Consequence** |
| --- | --- | --- | --- | --- | --- |
| P-01 |  |  |  |  |  |
| P-02 |  |  |  |  |  |

Facilitator instructions: Ask participants to identify the affected group, service context, underlying cause, and consequence before finalizing the problem statement. Check that the statement reflects an identified problem rather than a proposed solution.

**Template K2b. Weighted MCDA scoring matrix**

| **Candidate problem** | **Criterion 1 score** | **Criterion 2 score** | **Criterion 3 score** | **Criterion 4 score** | **Weighted total** | **Rank** | **Decision** |
| --- | --- | --- | --- | --- | --- | --- | --- |
| Problem 1 |  |  |  |  |  |  |  |
| Problem 2 |  |  |  |  |  |  |  |

Facilitator instructions: Enter the actual criteria and weights before scoring. Explain the scoring scale, record the agreed scores, calculate weighted totals using the established formula, and check the calculations with participants. Record the final rank and the decision taken.

**3.3. Idea generation and feasibility–impact templates (K3)**

**Template K3a. Idea generation and clustering**

| **HMW question** | **Original idea** | **Thematic cluster** | **Consolidated concept** | **Target user** |
| --- | --- | --- | --- | --- |
| HMW-01 |  |  |  |  |
| HMW-02 |  |  |  |  |

Facilitator instructions: Record ideas before merging duplicates. Preserve the original idea records, document the rationale for combining ideas, and describe each consolidated concept in a way that makes its purpose clear.

**Template K3b. Feasibility–Impact assessment**

| **Solution concept** | **Impact rating (1–5)** | **Feasibility rating (1–5)** | **Matrix quadrant** | **Selection rationale** |
| --- | --- | --- | --- | --- |
| Concept A |  |  |  |  |
| Concept B |  |  |  |  |

Facilitator instructions: Explain the rating criteria and scales before participants score concepts. Record the ratings, plot each concept according to the agreed method, and document why it was selected, deferred, or excluded from prototyping.

**3.4. Prototype review and role-play debrief form (K4)**

**Template K4. Prototype simulation debrief**

| **Prototype component** | **Scenario tested** | **Observed problem** | **User/provider feedback** | **Proposed modification** | **Final decision** |
| --- | --- | --- | --- | --- | --- |
|  |  |  |  |  |  |
|  |  |  |  |  |  |

Facilitator instructions: Explain the scenario, assign roles, observe the simulation, and record specific problems. After the simulation, invite participants to explain their experiences and discuss modifications. Record the final decision and its rationale.

**3.5. Supportive supervision and implementation monitoring (K5)**

**Template K5. Supportive supervision monitoring form**

| **Facility identifier** | **Component assessed** | **Fidelity assessment** | **Observed barrier** | **Corrective or supportive action** | **Follow-up status** |
| --- | --- | --- | --- | --- | --- |
|  |  | Yes / Partial / No / Not assessed |  |  |  |

Facilitator instructions: Record the component assessed, the evidence observed, and any implementation gaps. Distinguish observed findings from recommendations, agree on appropriate supportive actions with the responsible personnel, and document follow-up where available.

**3.6. Component refinement matrix and HCD decision log (K6–K7)**

**Template K6. Component refinement matrix**

| **Component** | **Field evidence** | **Strengths** | **Implementation challenge** | **Decision** | **Rationale** |
| --- | --- | --- | --- | --- | --- |
|  |  |  |  | Retain / Modify / Add / Remove / Clarify / Adapt |  |

Facilitator instructions: Review each component using the available field evidence and stakeholder feedback. Record the decision, its rationale, and any required modification. Avoid recording a proposed change as completed until the revised component has been verified.

**Template K7. HCD decision and adaptation log**

| **HCD phase** | **Date** | **Issue or stakeholder input** | **Stakeholder source** | **Decision** | **Resulting modification** | **Status** |
| --- | --- | --- | --- | --- | --- | --- |
|  |  |  |  |  |  | Proposed / Approved / Implemented |

The decision log should provide an auditable record of how stakeholder feedback and field-testing findings informed the final intervention package.

# PART C. WORKSHOP AGENDAS, SCHEDULES, AND OPERATIONAL PLANS

## 1. Master Schedule and Venue Overview

Table C1. Overview of HCD workshops, training activities, and field testing

| **Activity** | **HCD phase** | **Venue** | **Duration** | **Main participant groups** | **Principal output** |
| --- | --- | --- | --- | --- | --- |
| Workshop 1 | Empathize | Alliance Frugal Innovation Center | Full day; verify actual duration | Adolescents, providers, managers, officials | Empathy maps, journey maps, and identified barriers |
| Workshop 2 | Define | Alliance Frugal Innovation Center | Full day; verify actual duration | Adolescents, providers, managers, officials | Prioritized problems and HMW questions |
| Workshop 3 | Ideate | Alliance Frugal Innovation Center | Full day; verify actual duration | Adolescents, providers, managers, technical experts | Solution ideas and prioritized concepts |
| Workshop 4 | Prototype | CMHS, CPD Training room of AMU | Full day; verify actual duration | Design team, providers, managers, youth representatives, and relevant peer navigators | Prototypes and operational workflows |
| Training Stage 1 | Implementation preparation | CMHS, CPD Training room of AMU | Three days | Healthcare providers and facility managers | Preparation for assigned implementation roles |
| Training Stage 2 | Peer navigator preparation | CMHS, CPD Training room of AMU | Three days | Three selected trainers and adolescent/youth peer navigators | Preparation of peer navigators for field testing |
| Field testing | Test and Refine | Four participating primary healthcare facilities | Actual field-testing period | Implementers, peer navigators, and adolescent service users as applicable | Field-testing findings and supervision records |
| Workshop 5 | Test and Refine | CMHS, CPD Training room of AMU | Full day; verify actual duration | Adolescent representatives, providers, managers, officials, and other relevant stakeholders | Refinement decisions and final intervention package |

## 2. Detailed Time-Stamped Workshop Agendas

The agendas below retain the proposed 08:30–17:00 framework. Confirm the actual activity timings before treating them as a record of the workshops conducted.

### 2.1. Agenda 1: Empathize Workshop

Venue: Alliance Frugal Innovation Center
Participants: Project lead, design team, adolescent representatives, healthcare providers, facility managers, and district health officials.

| **Time** | **Activity** | **Purpose and method** | **Materials** | **Expected output** |
| --- | --- | --- | --- | --- |
| 08:30–09:00 | Registration and welcome | Participant registration and orientation | Attendance sheet, name tags | Registered participants |
| 09:00–09:30 | HCD orientation | Introduction to HCD principles and objectives | Slides, flipcharts | Shared understanding |
| 09:30–10:30 | Formative evidence review | Presentation and clarification of existing findings | Summary tables, projector | Shared evidence baseline |
| **10:30–10:45** | **Morning break** | **Break** | **—** | **—** |
| 10:45–12:00 | Stakeholder reflection | Facilitated experience sharing | Discussion prompts, flipcharts | Validated experience statements |
| 12:00–13:00 | Service gaps discussion | Identification of unmet needs and contextual barriers | Sticky notes, markers | Preliminary service-gap list |
| **13:00–14:00** | **Lunch break** | **Break** | **—** | **—** |
| 14:00–15:15 | Empathy mapping | Group mapping across six domains | Empathy templates | Completed empathy maps |
| 15:15–16:00 | Journey mapping | Mapping service experiences and barriers | Journey-map templates | Journey maps |
| 16:00–16:40 | Plenary synthesis | Consolidation and review of findings | Wall display | Consolidated barriers |
| 16:40–17:00 | Reflection and closure | Review of outputs and transition to Define | Summary sheet | Documented workshop outputs |

### 2.2. Agenda 2: Define Workshop

Venue: Alliance Frugal Innovation Center
Participants: Project lead, design team, adolescents, healthcare providers, facility managers, and district officials.

| **Time** | **Activity** | **Purpose and method** | **Materials** | **Expected output** |
| --- | --- | --- | --- | --- |
| 08:30–09:00 | Review of Empathize outputs | Revisit and validate identified barriers | Workshop summary | Reviewed barrier list |
| 09:00–10:00 | Affinity mapping | Group related barriers into themes | Sticky notes, wall tape | Thematic clusters |
| **10:00–10:15** | **Morning break** | **Break** | **—** | **—** |
| 10:15–11:15 | Problem definition | Develop structured problem statements | Problem-framing sheets | Candidate problem statements |
| 11:15–12:30 | MCDA criteria setting | Agree on criteria and weights | MCDA template | Documented criteria and weights |
| **12:30–13:30** | **Lunch break** | **Break** | **—** | **—** |
| 13:30–14:45 | Weighted MCDA scoring | Score and rank candidate problems | Scoring matrix | Ranked problem list |
| 14:45–15:30 | Ranking validation | Review scores and verify decisions | Calculation sheet | Validated priorities |
| **15:30–15:45** | **Afternoon break** | **Break** | **—** | **—** |
| 15:45–16:30 | HMW formulation | Convert prioritized problems into design questions | HMW worksheets | Draft HMW questions |
| 16:30–17:00 | Final validation | Review and agree on the outputs | Summary matrix | Finalized design questions |

### 2.3. Agenda 3: Ideate Workshop

Venue: Alliance Frugal Innovation Center
Participants: Project lead, design team, adolescents, healthcare providers, facility managers, and relevant technical experts.

| **Time** | **Activity** | **Purpose and method** | **Materials** | **Expected output** |
| --- | --- | --- | --- | --- |
| 08:30–09:00 | HMW review | Revisit the design questions | HMW cards | Shared design challenges |
| 09:00–09:30 | Ideation orientation | Explain brainstorming principles | Flipcharts | Shared ground rules |
| 09:30–10:30 | Individual brainstorming | Generate ideas independently | Sticky notes | Initial idea pool |
| **10:30–10:45** | **Morning break** | **Break** | **—** | **—** |
| 10:45–12:00 | Group brainstorming | Expand and combine ideas | Group flipcharts | Expanded solution pool |
| 12:00–13:00 | Idea clustering | Group related ideas and identify duplicates | Wall boards, tape | Thematic clusters |
| **13:00–14:00** | **Lunch break** | **Break** | **—** | **—** |
| 14:00–15:00 | Concept refinement | Consolidate ideas into solution concepts | Concept templates | Consolidated concepts |
| **15:00–15:15** | **Afternoon break** | **Break** | **—** | **—** |
| 15:15–16:00 | Feasibility–Impact assessment | Assess concepts using the agreed matrix | Matrix template | Assessed concepts |
| 16:00–16:40 | Concept selection | Review and prioritize concepts | Selection matrix | Shortlist for prototyping |
| 16:40–17:00 | Architecture synthesis | Integrate selected concepts into a preliminary package | Architecture template | Preliminary intervention structure |

### 2.4. Agenda 4: Prototype Workshop

Venue: CMHS, CPD Training Room, AMU
Participants: Design team, healthcare providers, facility managers, peer navigators, and adolescent representatives, as applicable.

| **Time** | **Activity** | **Purpose and method** | **Materials** | **Expected output** |
| --- | --- | --- | --- | --- |
| 08:30–09:00 | Concept recap | Review selected concepts | Ideation summary | Confirmed objectives |
| 09:00–09:30 | Prototyping orientation | Introduce rapid, low-fidelity prototyping | Sample tools | Shared understanding |
| 09:30–11:00 | Prototype development | Create preliminary intervention materials | Paper, markers, templates | Initial prototypes |
| **11:00–11:15** | **Morning break** | **Break** | **—** | **—** |
| 11:15–12:30 | Workflow mapping | Map service delivery and referral processes | Flowchart templates | Draft workflows |
| **12:30–13:30** | **Lunch break** | **Break** | **—** | **—** |
| 13:30–14:30 | Tool mock-up | Develop job aids, counseling materials, and relevant procedures | Draft tool sheets | Preliminary tools |
| 14:30–15:15 | Role-play simulation | Test service scenarios | Scenario cards | Usability observations |
| **15:15–15:30** | **Afternoon break** | **Break** | **—** | **—** |
| 15:30–16:15 | Prototype critique | Review usability and workflow gaps | Review checklist | Documented gaps |
| 16:15–16:45 | Rapid revision | Revise preliminary prototypes | Prototype materials | Revised prototypes |
| 16:45–17:00 | Field-testing preparation | Review outstanding preparation needs | Readiness checklist | Documented preparation actions |

### 2.5. Agenda 5: Implementer Training

**Venue:** CMHS, CPD Training Room, AMU
**Participants:** Healthcare providers and facility managers

**Number of days:** three days

| **Day** | **Training focus** | **Key activities** | **Expected output** |
| --- | --- | --- | --- |
| Day 1 | ASRH orientation and intervention rationale | ASRH principles, HCD rationale, and intervention components | Understanding of the intervention |
| Day 2 | Service delivery and peer navigation | Adolescent-responsive communication, privacy, confidentiality, and service-flow simulation | Familiarity with service procedures |
| Day 3 | Practical implementation preparation | Tool practice, scenario troubleshooting, and facility-specific planning | Preparation for assigned responsibilities |

### 2.6. Agenda 6: Peer Navigator Training

**Venue:** CMHS, CPD Training Room, AMU
**Participants:** Peer Navigators (Youth and Adolescents)

**Number of days:** three days

**Trainers:** Three selected participants from the initial training.

| **Day** | **Training focus** | **Key activities** | **Expected output** |
| --- | --- | --- | --- |
| Day 1 | Peer navigation roles | Responsibilities, intervention overview, and supporting adolescent access | Understanding of the peer navigator role |
| Day 2 | Communication and referral | Respectful communication, confidentiality, and referral pathways | Practical communication and navigation skills |
| Day 3 | Simulation and practical application | Scenario-based exercises and use of relevant intervention tools | Preparation for peer navigation activities |

Field-testing period: The intervention package was subsequently tested in four participating primary healthcare facilities. Implementation experiences and supervision records informed the subsequent stakeholder feedback and refinement process.

### 2.7. Agenda 6: Test and Refine Stakeholder Workshop

Venue: CPD Training Room, Arba Minch University.
Time: 08:30–17:00
Participants: Project lead, design team, adolescent representatives, healthcare providers, facility managers, district officials, and other relevant stakeholders.

| **Time** | **Activity** | **Purpose and method** | **Materials** | **Expected output** |
| --- | --- | --- | --- | --- |
| 08:30–09:00 | Welcome and opening | Establish objectives and ground rules | Attendance sheet | Shared expectations |
| 09:00–10:00 | Field-testing evidence review | Present available implementation and supervision findings | Slides, summary sheets | Shared evidence baseline |
| **10:00–10:15** | **Morning break** | **Break** | **—** | **—** |
| 10:15–11:15 | User feedback | Review user experience and usability | Feedback templates | Usability findings |
| 11:15–12:15 | Operational feasibility | Review workflow, workload, and contextual fit | Feasibility templates | Feasibility findings |
| 12:15–13:15 | Supervision synthesis | Review supervision findings and outstanding barriers | Supervision summaries | Consolidated implementation issues |
| **13:15–14:15** | **Lunch break** | **Break** | **—** | **—** |
| 14:15–15:15 | Component-by-component review | Assess intervention components using the refinement matrix | Refinement templates | Proposed refinement decisions |
| **15:15–15:30** | **Afternoon break** | **Break** | **—** | **—** |
| 15:30–16:15 | Workflow refinement | Review and revise relevant operational workflows | Workflow templates | Refined workflows |
| 16:15–16:45 | Package verification | Review the integrated intervention package | Package summary | Agreed final package |
| 16:45–17:00 | Decision-log completion | Document and verify final decisions | HCD Decision Log | Updated decision record |
