# Supplementary material for: Co-designing an integrated intervention package to strengthen adolescent sexual and reproductive health services in primary healthcare units in Southern Ethiopia: A human-centered design approach
Source: PLOS Glob Public Health. 2026 Nov 11;6(11):e0007554. doi: 10.1371/journal.pgph.0007554 (PMC13754124; doi:10.1371/journal.pgph.0007554)
Supplement: Supplementary file 2 [file pgph.0007554.s003.docx]

**S3 Appendix. Consolidated list and classification of 57 unique barriers**

| **No.** | **Broad domain** | **Barrier level** | **Barrier theme** | **Specific barrier** |
| --- | --- | --- | --- | --- |
| 1 | Individual and interpersonal | Intrapersonal | Knowledge and awareness | Limited awareness of available SRH services |
| 2 | Individual and interpersonal | Intrapersonal | Knowledge and awareness | Limited awareness of where SRH services are available |
| 3 | Individual and interpersonal | Intrapersonal | Knowledge and awareness | Limited comprehensive SRH education |
| 4 | Individual and interpersonal | Intrapersonal | Information access | Limited access to credible SRH information through digital and social media |
| 5 | Individual and interpersonal | Intrapersonal | Knowledge and misconceptions | Myths and misconceptions about SRH |
| 6 | Individual and interpersonal | Intrapersonal | Knowledge and misconceptions | Misconceptions about contraceptive methods |
| 7 | Individual and interpersonal | Intrapersonal | Psychological | Fear of judgment by healthcare providers |
| 8 | Individual and interpersonal | Intrapersonal | Psychological | Fear of judgment or stigma from community members |
| 9 | Individual and interpersonal | Intrapersonal | Psychological | Low self-confidence in seeking SRH services |
| 10 | Individual and interpersonal | Intrapersonal | Psychological | Shame, embarrassment, or guilt associated with SRH service utilization |
| 11 | Individual and interpersonal | Intrapersonal | Social perceptions | Perception that SRH services are intended only for married individuals |
| 12 | Individual and interpersonal | Intrapersonal | Social perceptions | Perception that seeking SRH services indicates promiscuity |
| 13 | Individual and interpersonal | Intrapersonal | Financial access | Inability to afford SRH services |
| 14 | Individual and interpersonal | Interpersonal | Family communication | Limited communication about SRH with parents or caregivers |
| 15 | Individual and interpersonal | Interpersonal | Family support | Unsupportive family environment |
| 16 | Individual and interpersonal | Interpersonal | Family reactions | Fear of negative parental or family reactions |
| 17 | Individual and interpersonal | Interpersonal | Family support | Family disapproval of SRH service utilization |
| 18 | Individual and interpersonal | Interpersonal | Family communication | Discomfort discussing SRH issues with parents or caregivers |
| 19 | Family and community | Community | Social stigma | Community stigma surrounding adolescent SRH service utilization |
| 20 | Family and community | Community | Social norms | Association of SRH service utilization with promiscuity |
| 21 | Family and community | Community | Social norms | Taboos surrounding adolescent sexuality |
| 22 | Family and community | Community | Social stigma | Fear of name-calling or being exposed when seeking SRH services |
| 23 | Family and community | Community | Gender norms | Gender norms limiting girls’ autonomy in SRH decision-making |
| 24 | Family and community | Community | Social stigma | Stigma contributing to delayed care-seeking or unsafe abortion choices |
| 25 | Family and community | Community | Social support | Limited supportive peer or community networks for SRH care-seeking |
| 26 | Family and community | Religious | Religious norms | Religious opposition to contraceptive use |
| 27 | Family and community | Religious | Religious norms | Religious opposition to SRH education |
| 28 | Family and community | Religious | Religious norms | Low acceptance of SRH services for unmarried adolescents |
| 29 | Health-system and structural | Health-system | Provider capacity | Inadequate provider competency in adolescent-responsive SRH care |
| 30 | Health-system and structural | Health-system | Provider training | Inadequate training in adolescent-responsive SRH service provision |
| 31 | Health-system and structural | Health-system | Provider attitudes | Judgmental provider attitudes toward adolescents |
| 32 | Health-system and structural | Health-system | Provider behavior | Stigmatizing or blaming adolescents seeking SRH services |
| 33 | Health-system and structural | Health-system | Confidentiality | Breach of confidentiality or disclosure of adolescents’ SRH information |
| 34 | Health-system and structural | Health-system | Service eligibility | Denial or restriction of services based on age |
| 35 | Health-system and structural | Health-system | Service eligibility | Requirement for parental consent to obtain SRH services |
| 36 | Health-system and structural | Facility | Service environment | Unfriendly or unwelcoming service environment |
| 37 | Health-system and structural | Facility | Privacy | Inadequate privacy during consultation and service provision |
| 38 | Health-system and structural | Facility | Adolescent responsiveness | Lack of adolescent-friendly spaces |
| 39 | Health-system and structural | Facility | Infrastructure | Overcrowded or poorly ventilated service areas |
| 40 | Health-system and structural | Facility | Adolescent services | Non-functional or poorly functioning youth-friendly centers |
| 41 | Health-system and structural | Infrastructure | Consultation space | Absence of adolescent-dedicated consultation rooms |
| 42 | Health-system and structural | Infrastructure | Service organization | Use of shared or multipurpose service areas |
| 43 | Health-system and structural | Accessibility | Service hours | Inconvenient service hours |
| 44 | Health-system and structural | Accessibility | Waiting time | Long waiting times for SRH services |
| 45 | Health-system and structural | Accessibility | Geographic access | Poor geographic access to SRH services |
| 46 | Health-system and structural | Accessibility | Transportation | Transportation difficulties |
| 47 | Health-system and structural | Commodities | Commodity availability | Frequent stockouts of essential SRH commodities |
| 48 | Health-system and structural | Information materials | IEC materials | Lack of adolescent-appropriate SRH information, education, and communication materials |
| 49 | Health-system and structural | Service delivery | Referral and linkage | Weak linkages between schools, communities, and health facilities |
| 50 | Health-system and structural | Service delivery | Clinical protocols | Inconsistent application of adolescent-responsive protocols |
| 51 | Health-system and structural | Service delivery | Service model | Predominantly reactive or case-based service delivery |
| 52 | Health-system and structural | Service delivery | Outreach | Limited or absent adolescent-focused outreach services |
| 53 | Health-system and structural | Governance | Coordination | Weak intersectoral collaboration and coordination |
| 54 | Health-system and structural | Governance | Accountability | Inadequate monitoring, evaluation, and accountability mechanisms |
| 55 | Health-system and structural | Governance | Participation | Limited involvement of adolescents in service planning and decision-making |
| 56 | Health-system and structural | Sustainability | Program continuity | Discontinuation of externally supported adolescent SRH programs without adequate replacement |
| 57 | Health-system and structural | Policy and financing | Policy and resources | Weak policy implementation, limited institutional and political commitment, and inadequate financing for adolescent-responsive SRH services |
