# Supplementary material for: Co-designing an integrated intervention package to strengthen adolescent sexual and reproductive health services in primary healthcare units in Southern Ethiopia: A human-centered design approach
Source: PLOS Glob Public Health. 2026 Nov 11;6(11):e0007554. doi: 10.1371/journal.pgph.0007554 (PMC13754124; doi:10.1371/journal.pgph.0007554)
Supplement: Supplementary file 3 [file pgph.0007554.s004.docx]

**S4 Appendix. Detailed Description of the Final Integrated Adolescent-Responsive Sexual and Reproductive Health Intervention Package**

The final integrated intervention package comprised four complementary components developed through iterative co-design, prioritization, prototyping, pilot testing, and refinement. The package was designed to address interconnected barriers to adolescents’ access to and utilization of SRH services within routine PHC settings. Rather than establishing a parallel service-delivery system, the package sought to strengthen existing PHC structures by improving provider capacity, facility responsiveness, adolescent awareness and navigation, and confidential access to information and referral support.

**Table A. Components, objectives, key activities, delivery mechanisms, and implementation responsibilities of the final integrated intervention package**

| **No.** | **Intervention component** | **Barriers addressed and objective** | **Key activities and operational procedures** | **Target users/beneficiaries** | **Delivery mechanism** | **Implementation responsibility and integration** |
| --- | --- | --- | --- | --- | --- | --- |
| **1** | **Capacity strengthening of healthcare providers and facility managers** | **Barriers:** Judgmental provider attitudes, limited adolescent-responsive competencies, inadequate communication and counseling skills, and concerns about privacy and confidentiality. **Objective:** Strengthen providers’ and facility managers’ capacity to deliver respectful, confidential, non-judgmental, client-centered, and adolescent-responsive SRH services. | • Conduct initial training and orientation on adolescent-responsive SRH service delivery and adolescent SRH rights and needs. • Incorporate VCAT to address provider attitudes and promote non-judgmental care. • Strengthen communication, counseling, confidentiality, privacy protection, and client-centered service-delivery skills. • Provide mentorship, refresher sessions, and supportive supervision. • Use structured performance feedback to identify service-delivery gaps and reinforce good practices. • Provide relevant job aids and service-delivery materials to support routine practice. | Healthcare providers, including staff providing SRH services, and facility managers. Adolescents benefit through improved quality and experience of care. | Initial training, on-site orientation, mentorship, refresher sessions, supportive supervision, job aids, and structured performance feedback integrated into routine PHC operations. | The research/project team supports initial capacity strengthening and implementation. Facility managers, trained providers, and designated focal persons support ongoing mentorship, supervision, performance feedback, and integration into routine services. |
| **2** | **Adolescent-responsive health facility environment** | **Barriers:** Inadequate privacy, uncertainty about confidentiality, intimidating service environments, unnecessary waiting, limited awareness of available services, and insufficient adaptation of routine services to adolescent needs. **Objective:** Improve the accessibility, acceptability, privacy, confidentiality, and responsiveness of PHC facilities and create a more welcoming environment for adolescents seeking SRH services. | • Introduce practical measures to improve privacy and confidentiality during service encounters. • Review service organization and patient flow to reduce unnecessary waiting and improve access to appropriate services. • Promote respectful, inclusive, and adolescent-responsive interactions throughout the facility. • Display banners, educational leaflets, and other visual communication materials to explain available SRH services and communicate messages about confidentiality, inclusiveness, and respectful care. • Improve the organization and presentation of adolescent-relevant service information and directions. • Identify facility-level barriers and implement feasible service improvements through routine monitoring and quality improvement. | Adolescents and young people accessing PHC services; healthcare providers, facility managers, and other relevant facility personnel responsible for service organization. | Facility-based environmental and organizational adaptations, visual communication materials, improved patient flow, and continuous quality improvement embedded in routine PHC delivery. | Facility managers, healthcare providers, and designated facility focal persons lead implementation and routine monitoring, with technical support from the research/project team and relevant district health stakeholders as appropriate. |
| **3** | **Integrated adolescent peer navigation system** | **Barriers:** Limited awareness of available SRH services, uncertainty about where to obtain care, limited trust in formal services, difficulties approaching facilities independently, and weak referral and linkage pathways. **Objective:** Connect adolescents with trusted SRH information and basic counseling, facilitate navigation to appropriate services, and strengthen referral, linkage, and continuity of care. | • Identify, select, and train adolescent peer navigators in SRH information provision, basic counseling within their defined scope, confidentiality, communication, referral, and navigation. • Provide accurate, age-appropriate SRH information through community outreach, adolescent gathering points, and facility-linked activities. • Help adolescents understand available services and how to access them. • Identify adolescents who may benefit from professional SRH services and facilitate referrals without undertaking clinical diagnosis or care beyond the navigator’s role. • Provide navigation and referral assistance to appropriate PHC facilities and other relevant support services. • Coordinate with designated facility focal persons and support referral follow-up and linkage, subject to adolescents’ consent and appropriate confidentiality safeguards. • Monitor referrals and identify gaps in linkage to inform service improvement. | Adolescents and young people, particularly those facing informational, social, trust-related, or service-navigation barriers to SRH access. | Peer-to-peer engagement, community outreach, adolescent gathering points, facility-linked activities, supported referrals, navigation, and follow-up through established PHC referral pathways. | Trained adolescent peer navigators operate within clearly defined roles and under the supervision of designated facility focal persons and facility managers, with support from the research/project team and relevant community stakeholders. Healthcare providers receive referred adolescents and provide appropriate clinical services. |
| **4** | **Confidential SRH call center and linkage support** | **Barriers:** Concerns about stigma and privacy, difficulties obtaining reliable SRH information, reluctance to approach facilities directly, and challenges connecting adolescents with appropriate facility-based services. **Objective:** Provide an additional confidential access pathway for SRH information and counseling and facilitate adolescents’ linkage to appropriate PHC services. | • Establish or strengthen a telephone-based mechanism for confidential SRH information and counseling, subject to operational feasibility. • Provide standardized, accurate, age-appropriate information using adolescent-friendly and non-judgmental communication. • Respond to questions concerning contraception, menstrual health, sexually transmitted infections, pregnancy-related concerns, and other SRH topics within the service’s scope. • Provide appointment information or support where available and appropriate. • Identify callers who may require clinical assessment or other specialized support and facilitate referral to appropriate services. • Document calls and referrals using secure procedures that protect confidentiality. • Develop referral and follow-up procedures to support linkage with PHC facilities and other appropriate services. • Monitor service operation and referral processes if the mechanism is implemented. | Adolescents and young people seeking remote, confidential SRH information, counseling, appointment support, or referral assistance. | Telephone-based information provision and counseling, with referral, appointment support, and linkage to existing facility-based services. | Trained healthcare providers or qualified counselors would provide the service, with oversight from designated service coordinators and facility managers. Implementation would require appropriate staffing, secure documentation, confidentiality safeguards, and established referral arrangements. The operational status of the call center should be reported separately from the other components. |
