# Supplementary material for: Co-designing an integrated intervention package to strengthen adolescent sexual and reproductive health services in primary healthcare units in Southern Ethiopia: A human-centered design approach
Source: PLOS Glob Public Health. 2026 Nov 11;6(11):e0007554. doi: 10.1371/journal.pgph.0007554 (PMC13754124; doi:10.1371/journal.pgph.0007554)
Supplement: Supplementary file 4 [file pgph.0007554.s005.docx]

**S5 Appendix. Overview of the Human-Centered Design Co-Design Process for Developing the Adolescent-Responsive Primary Healthcare Intervention in South Ethiopia**

The human-centered design co-design process followed five iterative phases—Empathize, Define, Ideate, Prototype, and Test supported by continuous stakeholder engagement, evidence synthesis, and cross-phase refinement. The process progressed from understanding adolescents’ experiences and the primary healthcare context to identifying and prioritizing implementation challenges, generating and selecting potential solutions, developing intervention prototypes, and refining the final integrated intervention package through pilot implementation and stakeholder feedback.

| **HCD phase** | **Primary objective** | **Participants and stakeholders** | **Key activities and methods** | **Data and evidence generated** | **Main outputs and design decisions** |
| --- | --- | --- | --- | --- | --- |
| **1. Empathize** | Understand adolescents’ experiences, needs, preferences, and barriers to accessing and utilizing SRH services, alongside provider, facility, community, and health-system perspectives on service delivery and implementation context. | Adolescents and young people; healthcare providers; facility managers; district health stakeholders; community representatives; and researchers. | • Conduct formative qualitative interviews and stakeholder consultations. • Assess facility-level service-delivery conditions and contextual factors. • Explore experiences and perceptions related to accessibility, awareness, privacy, confidentiality, provider attitudes, service responsiveness, and health-system readiness. • Synthesize stakeholder accounts and contextual evidence to identify barriers and facilitators. | Qualitative accounts, contextual observations, facility-level evidence, and stakeholder perspectives on barriers and facilitators affecting adolescent SRH service access and utilization. | Identified and classified **57 unique implementation challenges/barriers** across relevant contextual, individual, interpersonal, and service-delivery domains. The findings established the evidence base for subsequent problem prioritization. |
| **2. Define** | Synthesize the formative evidence to identify, prioritize, and clearly frame the most important and actionable implementation problems that could be addressed through a contextually appropriate intervention. | Adolescents and young people; healthcare providers; facility managers; district health officials; community representatives; and researchers. | • Review and synthesize barriers identified during the Empathize phase. • Conduct structured, participatory problem-definition and prioritization discussions. • Apply weighted Multi-Criteria Decision Analysis (MCDA) to assess candidate problems against the agreed prioritization criteria. • Formulate *How Might We* (HMW) questions to translate priority problems into user-centered opportunities for intervention design. | Consolidated barrier classifications, comparative problem-prioritization results, stakeholder judgments, and user-centered problem statements. | Prioritized **19 implementation problems** and formulated **10 HMW questions** to guide solution generation. The HMW questions provided the design focus for the Ideate phase. |
| **3. Ideate** | Generate a broad range of potential, stakeholder-informed solutions to the prioritized problems and identify promising concepts for further development. | Adolescents and young people; healthcare providers; facility managers; district health officials; community representatives; and researchers. | • Conduct structured brainstorming guided by the 10 HMW questions. • Encourage individual and group generation of potential solutions. • Cluster related ideas using affinity mapping or equivalent structured synthesis methods. • Review candidate ideas for relevance to the identified barriers and stakeholder needs. • Apply a feasibility–impact matrix to compare and prioritize promising intervention concepts. | Candidate intervention ideas, thematic groupings of related solutions, stakeholder assessments, and comparative feasibility–impact judgments. | Generated **140 intervention ideas**, which were clustered and screened to identify promising concepts. A feasibility–impact matrix supported the selection of high-priority ideas for prototyping. |
| **4. Prototype** | Translate selected solution concepts into practical, testable intervention components and specify how these components could operate within existing PHC structures. | Selected adolescents and young people; healthcare providers; facility managers; relevant implementers and stakeholders; and researchers. | • Develop low-fidelity prototypes and preliminary intervention workflows. • Define proposed activities, delivery mechanisms, stakeholder roles, and referral and linkage processes. • Use role-play, simulation, or other relevant methods to examine operational feasibility where applicable. • Review prototype concepts with stakeholders and assess their contextual relevance and compatibility with routine PHC services. • Incorporate feedback and refine the draft intervention package before pilot testing. | Prototype descriptions, proposed workflows, role and responsibility specifications, operational requirements, stakeholder feedback, and identified implementation considerations. | Developed a draft integrated intervention package with preliminary component descriptions, activities, delivery arrangements, and implementation responsibilities. The draft package was refined to address stakeholder concerns and operational requirements before testing. |
| **5. Test** | Examine the practical implementation of the draft intervention package, identify operational challenges, and incorporate implementation experience and stakeholder feedback into its refinement. | Implementers; healthcare providers; facility managers; adolescents and young people; and researchers. | • Conduct three-day implementer training. • Undertake a one-month pilot implementation in four purposively selected PHC facilities. • Provide supportive supervision and document implementation observations. • Gather feedback from relevant stakeholders and reflect on implementation experiences. • Identify operational challenges, feasible adaptations, and modifications needed to improve the intervention package. | Implementer and stakeholder feedback, implementation observations, operational challenges, contextual-fit assessments, and documented priorities for refinement. | Refined and finalized the intervention package based on pilot implementation experience and stakeholder feedback, with modifications intended to improve feasibility, usability, acceptability, and compatibility with routine PHC delivery. Pilot findings informed refinement but were not treated as definitive evidence of intervention effectiveness. |
| **Cross-phase iteration and integration** | Maintain continuity across the HCD phases, ensure that stakeholder perspectives inform successive design decisions, and integrate learning into a coherent intervention package. | Stakeholders engaged across the HCD phases, including adolescents and young people, healthcare providers, facility managers, community representatives, district health stakeholders, implementers, and researchers, as applicable to each activity. | • Review and synthesize evidence and feedback generated across phases. • Revisit emerging problems, candidate solutions, and prototype components as new information becomes available. • Incorporate stakeholder feedback and implementation learning into successive design iterations. • Document key refinements and integrate complementary intervention components within existing PHC structures. | Cross-phase synthesis, stakeholder feedback, documented design decisions, implementation learning, and successive versions of the intervention package. | Produced the final integrated **Adolescent-Responsive Primary Healthcare intervention package**, comprising four complementary components: (1) capacity strengthening of healthcare providers and facility managers; (2) an adolescent-responsive health facility environment; (3) an integrated adolescent peer navigation system; and (4) confidential SRH call-center and linkage support. The components were designed to address interconnected barriers to adolescents’ access to and utilization of SRH services within routine PHC settings. |
